# From Cognitive Impairment to Uremic Encephalopathy in Chronic Kidney Disease: A Proposed Pathophysiological Continuum

**DOI:** 10.3390/ijms27198768

**Published:** 2026-09-30

**Authors:** Jelena Nesovic Ostojic, Andrija Vukovic, Sanjin Kovacevic, Aleksandra Nenadovic, Danijela Karanovic, Silvio R. De Luka

**Affiliations:** 1 Department of Pathological Physiology, Faculty of Medicine, University of Belgrade, 11000 Belgrade, Serbia; jelena.nesovic-ostojic@med.bg.ac.rs (J.N.O.); andrija.vukovic@med.bg.ac.rs (A.V.); sanjin.kovacevic@med.bg.ac.rs (S.K.); aleksandra.nenadovic@med.bg.ac.rs (A.N.); 2 Department of Cardiovascular Physiology, Institute for Medical Research, National Institute of the Republic of Serbia, University of Belgrade, 11000 Belgrade, Serbia; danijela.karanovic@imi.bg.ac.rs

**Keywords:** chronic kidney disease, cognitive impairment, uremic encephalopathy, uremic toxins, blood–brain barrier, neuroinflammation, oxidative stress, mitochondrial dysfunction

## Abstract

Chronic kidney disease (CKD) is associated with neurological abnormalities ranging from subtle cognitive deficits to acute or subacute uremic encephalopathy. Although clinically distinct, these syndromes may arise from overlapping biological processes, including retained uremic solutes, cerebrovascular and blood–brain barrier dysfunction, systemic and neuroinflammation, oxidative stress, mitochondrial impairment, and altered neurotransmission. This narrative review synthesizes clinical, experimental, and cellular evidence supporting the contribution of these interconnected pathways to CKD-associated brain dysfunction. We propose that chronic cognitive impairment reflects progressively reduced neuronal and neurovascular reserves. In contrast, uremic encephalopathy may emerge when severe metabolic, neurotoxic, inflammatory, or hemodynamic stress exceeds the remaining capacity for cerebral adaptation. Uremic toxins may act as CKD-specific upstream drivers of endothelial injury, glial activation, redox imbalance, and cellular metabolic dysfunction. These processes converge on impaired mitochondrial bioenergetics, disrupted synaptic homeostasis, reduced cerebrovascular responsiveness, and increased neuronal vulnerability. However, direct longitudinal evidence for a linear progression from cognitive impairment to uremic encephalopathy remains limited. We therefore propose a pathophysiological continuum linking chronic cerebral vulnerability with acute uremic decompensation, intended as a mechanistic model rather than as a diagnostic or classification framework. Clarifying their shared and distinguishing mechanisms may improve recognition of neurological vulnerability and support earlier therapeutic targeting of the kidney–brain axis.

## 1. Introduction

Chronic kidney disease (CKD) is a major global public health challenge, affecting approximately 9–10% of the world’s population and representing a leading cause of morbidity, mortality, and disability worldwide. Despite advances in prevention and treatment, the global burden of CKD continues to increase owing to population aging and the rising prevalence of diabetes, hypertension, and other chronic diseases. Consequently, CKD has emerged as a major contributor to healthcare utilization and premature mortality, emphasizing the importance of strategies aimed at delaying disease progression and preventing systemic complications [1,2].

Neurological complications are increasingly recognized as important manifestations of CKD and may occur throughout the disease course, from early renal dysfunction to end-stage kidney disease (ESKD), involving both the central and peripheral nervous systems [3,4,5,6]. Among these, cognitive impairment and uremic encephalopathy (UE) represent the most clinically significant forms of CKD-related brain dysfunction because of their high prevalence, association with adverse clinical outcomes, and profound impact on functional independence and quality of life. Cognitive impairment affects approximately 20–50% of patients with moderate CKD and up to 70% of older adults receiving maintenance dialysis, although reported prevalence varies according to patient characteristics and the cognitive assessment methods employed [7,8,9].

Cognitive deficits predominantly involve attention, executive function, processing speed, memory, and visuospatial abilities and may be detectable even in the early stages of CKD [10]. Importantly, cognitive impairment remains markedly underrecognized in routine clinical practice despite being associated with adverse clinical outcomes, including reduced treatment adherence, hospitalization, frailty, dialysis withdrawal, and increased mortality [7,11].

UE is an acute or subacute neuropsychiatric syndrome of advanced kidney failure characterized by impaired attention, fluctuating consciousness, delirium, asterixis, seizures, and, in severe cases, coma. Neurological manifestations are often at least partially reversible after dialysis or another form of kidney replacement therapy, supporting a major contribution of functional metabolic and neurophysiological disturbance rather than primary irreversible neurodegeneration [12,13].

CKD-associated cognitive impairment and UE are clinically distinct syndromes, and current evidence does not establish that chronic cognitive impairment inevitably progresses to encephalopathy. Nevertheless, both conditions arise within a shared biological environment that includes uremic toxin retention, endothelial and cerebral small-vessel injury, blood-brain barrier (BBB) disruption, systemic inflammation and neuroinflammation, oxidative stress, mitochondrial dysfunction, and altered neurotransmission [14,15]. Although recent reviews have addressed cognitive decline in CKD and the clinical and pathophysiological features of UE separately [12,14], the relationship between these syndromes remains incompletely defined. Accordingly, the present review examines the biological interface between chronic cognitive dysfunction and acute UE and proposes a non-linear reserve-threshold model in which cumulative neuronal and neurovascular injury may progressively reduce cerebral adaptive capacity, while superimposed uremic, metabolic, inflammatory, hemodynamic, or osmotic stress may precipitate acute encephalopathic decompensation. By integrating clinical, experimental, and cellular evidence, we also derive testable predictions concerning cerebral vulnerability and the emergence of overt UE. The proposed model is intended as a hypothesis-generating conceptual construct rather than as an established diagnostic or classification entity.

## 2. Review Scope and Literature Search

This narrative review examines the clinical and biological mechanisms that may connect chronic cognitive impairment with acute UE across the course of CKD. Evidence was considered at three complementary levels: clinical findings in patients with CKD or kidney failure, experimental observations from animal models, and toxin-specific or cell-specific effects demonstrated in vitro or ex vivo. Particular attention was given to the distinction between CKD-specific upstream drivers, including retained uremic solutes, and shared downstream pathways involving cerebrovascular and neurovascular dysfunction, BBB disruption, systemic and neuroinflammation, oxidative stress, mitochondrial impairment, and altered neurotransmission.

Relevant studies were identified through searches of PubMed/MEDLINE, Web of Science, and Google Scholar, last updated on 20 July 2026. The search focused primarily on publications from January 2016 to July 2026, while earlier studies were included when they represented seminal contributions or were essential for understanding CKD-associated neurological dysfunction. Search terms included combinations of “chronic kidney disease,” “kidney failure,” “uremia,” “uremic encephalopathy,” “cognitive impairment,” “brain dysfunction,” “uremic toxins,” “blood–brain barrier,” “neurovascular dysfunction,” “neuroinflammation,” “oxidative stress,” “mitochondrial dysfunction,” “synaptic dysfunction,” “dialysis,” and “kidney replacement therapy.” Additional searches addressed individual retained solutes and mediators, including indoxyl sulfate, p-cresyl sulfate, guanidino compounds, phenylacetylglutamine, and parathyroid hormone.

Clinical observational and interventional studies, experimental animal studies, and in vitro or ex vivo investigations were considered according to their relevance to CKD-associated cognitive or neurological dysfunction and the proposed pathophysiological framework. Of the 167 references cited in the final manuscript, 63 were review articles, 2 were other secondary publications, and no book chapters were included; the remaining 102 references reported original data. These secondary sources were used primarily to provide context and to identify additional relevant primary studies, while original studies were preferentially cited for specific clinical and mechanistic findings whenever available. Because this article was designed as a narrative and concept-oriented review, literature identification and selection were iterative rather than being intended to achieve systematic-review exhaustiveness; no formal protocol registration, meta-analysis, or quantitative risk-of-bias assessment was performed.

## 3. CKD-Associated Brain Dysfunction: Distinct Clinical Syndromes with Shared Pathophysiology

**Figure 1:**
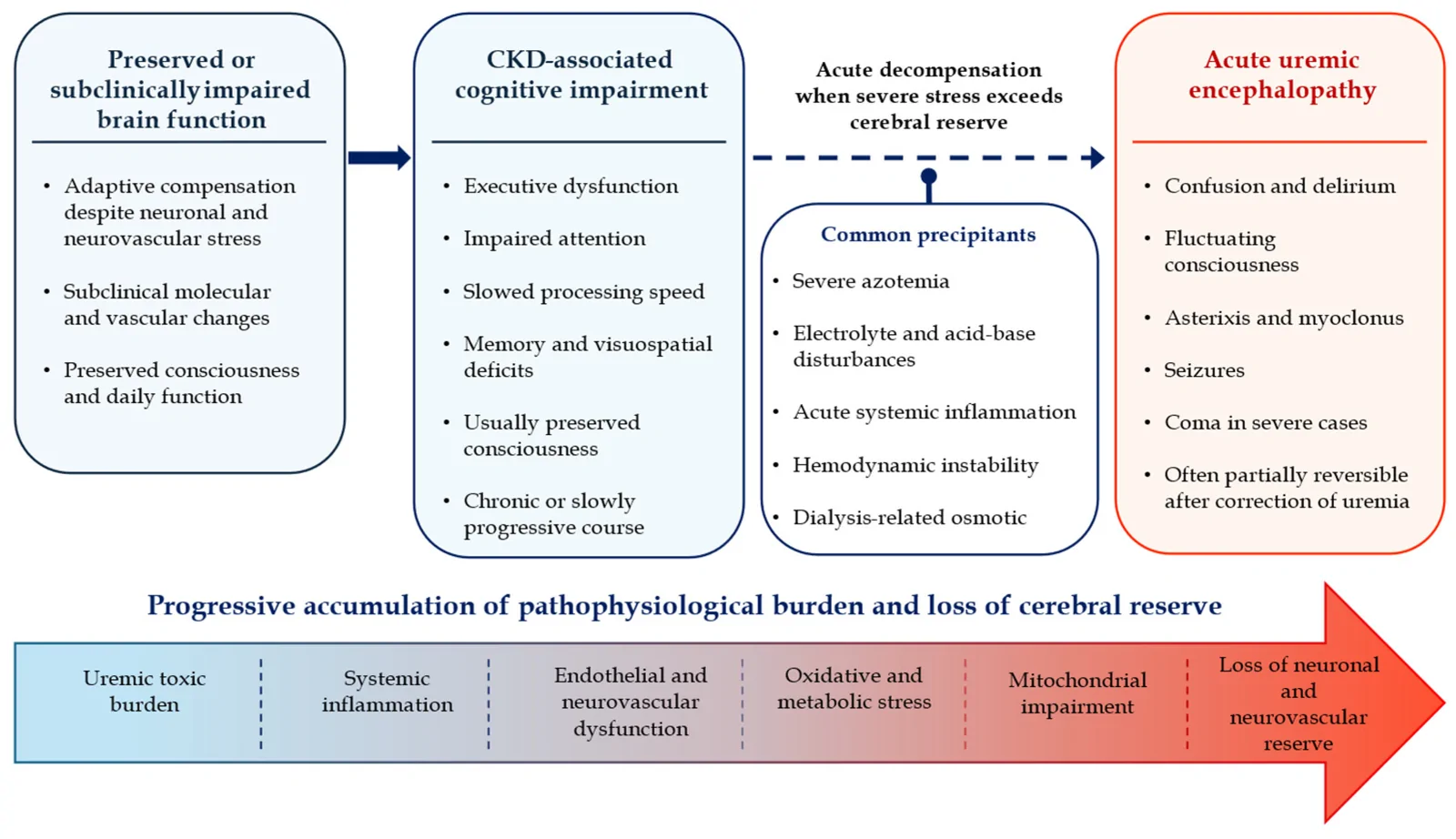
Proposed reserve–threshold model of CKD-associated brain dysfunction. CKD-associated brain dysfunction may range from preserved or subclinically impaired cerebral function to chronic cognitive impairment and, in advanced kidney failure, acute encephalopathic decompensation. Chronic CKD-related toxic, vascular, inflammatory, metabolic, and mitochondrial stress may progressively reduce neuronal and neurovascular reserve, while UE is proposed to emerge when acute uremic or systemic stress exceeds the remaining adaptive capacity. The dashed arrow indicates that progression from cognitive impairment to UE is proposed rather than inevitable. CKD, chronic kidney disease; UE, uremic encephalopathy.

CKD-associated brain dysfunction encompasses a broad clinical spectrum, ranging from slowly evolving, multidomain cognitive impairment to acute or subacute UE [7,12,16]. Chronic cognitive impairment may become detectable before advanced kidney failure and is commonly associated with cerebral small-vessel disease, white matter injury, neurovascular dysfunction, and other structural brain abnormalities [7,16]. By contrast, UE typically occurs in severe kidney failure or during acute uremic decompensation and presents with diffuse cerebral dysfunction, including confusion, delirium, impaired awareness, motor signs such as asterixis and myoclonus, epileptic manifestations, and, in severe cases, coma [12]. For clarity, the terminology used in this review reflects different levels of clinical classification. Uremic encephalopathy is an established clinical syndrome, whereas cerebral small-vessel disease (CSVD) is used here as an established umbrella term encompassing heterogeneous cerebral microvascular pathologies and not as a single standalone nosological category. In contrast, the pathophysiological continuum and reserve–threshold model proposed in this review are conceptual constructs intended to integrate the available evidence and should not be interpreted as established diagnostic or classification entities. Although the two syndromes may share several biological mechanisms, they remain clinically distinct, and progression from chronic cognitive impairment to UE is neither obligatory nor necessarily linear [12,17,18]. Neuroimaging and cerebrovascular studies further support the presence of structural and vascular cerebral vulnerability in CKD [19,20]. The following sections therefore examine these shared mechanisms as potential contributors to chronic cerebral vulnerability and acute decompensation rather than as evidence of a uniform chronological sequence (Figure 1).

## 4. Uremic Toxins as Upstream Drivers of CKD-Associated Brain Dysfunction

Accumulation of retained solutes is one of the strongest CKD-specific links between kidney dysfunction and cerebral injury. A wide range of compounds accumulate as kidney function declines, but current evidence does not support a single dominant neurotoxin. Instead, small water-soluble solutes, protein-bound toxins, and middle molecules contribute to a uremic milieu characterized by inflammatory activation, endothelial injury, altered metabolic signaling, and disruption of neurovascular homeostasis [21].

Gut microbiota-derived protein-bound toxins have received particular attention because they are incompletely removed by conventional dialysis and exert broad effects on vascular and neural tissues. Indoxyl sulfate (IS) is the most extensively investigated, but indole-3-acetic acid (IAA), p-cresyl sulfate (PCS), phenylacetylglutamine (PAG), β_2_-microglobulin, guanidino compounds, and other retained metabolites may make distinct and potentially synergistic contributions to CKD-associated brain dysfunction (Table 1, Table 2 and Table 3).

### 4.1. Clinical Evidence

In patients receiving maintenance hemodialysis, higher circulating IS concentrations have been independently associated with poorer global cognitive performance assessed using the Mini-Mental State Examination (MMSE), Cognitive Abilities Screening Instrument (CASI), and Montreal Cognitive Assessment (MoCA). Elevated IS levels have also been linked to impairment across multiple cognitive domains, including memory, language, mental manipulation, and visuospatial function [22,23]. Notably, associations between protein-bound uremic toxins and cognitive performance appear stronger than those observed for conventional markers of uremia, including blood urea nitrogen and creatinine, suggesting that the composition of the uremic milieu may be more relevant for neurological outcomes than the degree of azotemia alone [23]. Importantly, Wang et al. [23] also provided interventional evidence by randomizing 135 patients receiving maintenance hemodialysis to conventional hemodialysis (HD), hemodialysis combined with hemodiafiltration (HD + HDF), or hemodialysis combined with hemodiafiltration and hemoperfusion (HD + HDF + HP). Patients receiving the combined HD + HDF + HP regimen showed the greatest reductions in circulating homocysteine (Hcy) and IS concentrations, together with the greatest improvement in Montreal Cognitive Assessment (MoCA) scores. These findings support an association between broader removal of retained uremic solutes and improved cognitive performance. However, because randomization concerned dialysis modality rather than exposure to an individual uremic toxin, and because several retained solutes changed simultaneously, the study does not establish a toxin-specific causal effect of IS on cognitive function [23].

Recent clinical evidence further suggests that impaired neurovascular integrity may represent an important pathway connecting systemic uremia with cerebral dysfunction. Bobot et al. (2024) [18] demonstrated that patients with ESKD exhibited increased BBB permeability accompanied by higher circulating concentrations of IS, IAA, and PCS, enhanced aryl hydrocarbon receptor (AhR) activation, and poorer cognitive performance compared with healthy controls. Moreover, BBB permeability was inversely correlated with cognitive scores, supporting the concept that impaired barrier integrity may increase cerebral exposure to circulating uremic metabolites and enhance their neurotoxic potential. However, these findings remain primarily observational, and the temporal relationship between toxin accumulation and BBB dysfunction requires further clarification.

While IS has been implicated in CKD-associated neurotoxicity, clinical evidence indicates that additional retained metabolites may also contribute to cognitive impairment. IAA, another gut microbiota-derived protein-bound tryptophan metabolite, has been independently associated with impaired cognitive performance in patients undergoing maintenance hemodialysis. Higher serum IAA concentrations were associated with lower MMSE and CASI scores and an approximately twofold higher likelihood of cognitive impairment defined with these instruments [24]. In contrast, hippuric acid, which was evaluated in the same study, was not significantly associated with cognitive performance. Similarly, PCS has been linked to cognitive dysfunction, although reported associations appear less consistent and may depend on the cognitive domain evaluated [18,22]. These observations suggest that individual uremic metabolites may exert partially distinct effects on cerebral networks rather than producing uniform neurological abnormalities.

More recently, PAG has emerged as a potential neuroactive uremic metabolite. In the Chronic Kidney Disease-Renal Epidemiology and Information Network (CKD-REIN) cohort, including more than 2500 patients with CKD stages 2–5, higher serum PAG concentrations were independently associated with poorer cognitive performance. Each twofold increase in PAG levels corresponded to an approximately 12–15% higher adjusted risk of impaired MMSE scores [25]. These cross-sectional findings indicate that the association between higher PAG concentrations and cognitive impairment is present across CKD stages 2–5; however, they do not establish whether PAG elevation precedes cognitive decline. More broadly, although several of these studies used multivariable models to account for relevant demographic and clinical covariates, such adjustment cannot eliminate residual confounding. Therefore, the reported associations of IS, IAA, PCS, and PAG with cognitive outcomes should be interpreted cautiously, as kidney function, nutritional status, dialysis-related factors, and other clinical characteristics may influence both circulating toxin concentrations and cognitive performance.

Beyond individual toxins, the overall burden and clearance of retained solutes appear to influence cognitive outcomes. In patients receiving peritoneal dialysis, greater clearance of the middle molecule β_2_-microglobulin was associated with better global cognition, improved executive function, and a more favorable cognitive trajectory over two years. In contrast, conventional measures of small-solute clearance, including Kt/V (a measure of dialysis adequacy; K, dialyzer urea clearance; t, dialysis treatment time; V, urea distribution volume), and urea removal showed weaker associations [26]. These findings indicate that CKD-associated neurological consequences cannot be adequately explained by small-solute clearance alone.

Longitudinal studies further suggest that partial correction of the uremic milieu may improve cognitive performance. Initiation of dialysis has been linked with modest improvements in global cognition, particularly during the first year of treatment, indicating that some components of uremic brain dysfunction may be reversible. However, persistent executive deficits despite adequate dialysis may reflect incomplete removal of protein-bound toxins, together with chronic vascular and inflammatory alterations, may sustain ongoing neuronal dysfunction [27]. This limitation of current kidney replacement therapies is particularly relevant since many protein-bound uremic toxins remain poorly cleared by conventional dialysis.

These findings should, however, be interpreted with methodological caution. Common screening instruments, including the Mini-Mental State Examination (MMSE) and Montreal Cognitive Assessment (MoCA), were not specifically developed for patients undergoing dialysis, and cognitive performance may be influenced by the timing and conditions of assessment relative to a hemodialysis session. Studies comparing cognitive testing before, during, and after hemodialysis have demonstrated within-patient variability in performance, supporting the need for standardized testing conditions [28]. Therefore, differences in cognitive instruments, testing environment, and timing relative to dialysis should be considered when interpreting and comparing the clinical studies summarized in Table 1, and cognitive scores obtained under different dialysis conditions should not be regarded as directly interchangeable.

Although direct evidence linking IS to cognitive dysfunction outside CKD populations remains limited, observations from other clinical settings provide additional support for its neuroactive properties. In treatment-naive patients with major depressive disorder, higher circulating IS concentrations were associated with altered resting-state functional connectivity of the subcallosal cingulate cortex and greater anxiety severity. Changes in functional connectivity partially mediated the relationship between IS concentrations and psychiatric symptoms, suggesting that gut microbiota-derived metabolites may influence human brain network function independently of kidney disease [29].

**Table 1:** Clinical evidence linking retained uremic solutes to cognitive dysfunction in CKD, with supportive evidence from a non-CKD cohort.

Study	Study Design/Population	CKD Stage/Model	Uremic Toxin(s) Investigated	Cognitive/Neurological Assessment	Main Findings	Proposed Mechanism(s)
Levassort et al. (2025) [25]	Cross-sectional analysis (CKD-REIN cohort); 2590 patients	CKD stages 2–5	PAG, IS, PCS, TMAO	MMSE (cognitive impairment defined as ≤26)	Higher serum PAG concentrations were independently associated with cognitive impairment; each twofold increase in PAG was associated with an approximately 12–15% higher adjusted risk of impaired cognition	PAG represents a potential neuroactive gut-derived uremic metabolite; possible effects through neurovascular dysfunction and altered metabolic signaling
Bobot et al. (2024) [18]	Prospective comparative study; 15 ESKD patients on HD and 15 healthy controls	ESKD	IS, IAA, PCS; AhR activation	MoCA, Stroop test, Doors test, McNair questionnaire; BBB permeability assessed by 99mTc-DTPA SPECT/CT	ESKD patients exhibited increased BBB permeability, higher circulating toxin concentrations, enhanced AhR activation, and poorer cognitive performance; BBB permeability correlated inversely with cognitive scores	Protein-bound toxins promote BBB dysfunction through AhR activation, increasing CNS exposure to circulating metabolites and amplifying neuroinflammatory signaling
Lin et al. (2019) [22]	Cross-sectional study; 260 maintenance HD patients and 55 controls	ESKD on HD	Free IS and PCS	MMSE, CASI, CES-D	Higher IS concentrations were independently associated with lower MMSE and CASI scores and impaired memory, language, mental manipulation, and visuospatial function; PCS associations were weaker	Protein-bound toxins may contribute to cognitive dysfunction through oxidative stress, endothelial injury, and direct neuronal effects
Lin et al. (2019) [24]	Cross-sectional study; 230 HD patients	ESKD on HD	IAA, hippuric acid	MMSE, MoCA, CASI	Higher IAA concentrations were independently associated with lower cognitive scores and approximately twofold increased risk of cognitive impairment; hippuric acid showed no significant association	IAA may contribute to neuroinflammation, oxidative stress, and neuronal dysfunction
Wang et al. (2025) [23]	Randomized three-arm comparative interventional study; 135 maintenance HD patients (45 per group)	ESKD	IS, homocysteine, β_2_-microglobulin, iPTH, creatinine, BUN	MoCA	HD + HDF + HP produced the greatest reductions in Hcy and IS and the greatest improvement in MoCA scores; Hcy and IS were independently associated with cognitive impairment.	Protein-bound toxin accumulation may promote oxidative stress, endothelial dysfunction, and impaired cerebral homeostasis
Li et al. (2019) [26]	Prospective cohort; 222 peritoneal dialysis patients (130 followed longitudinally for 2 years)	CKD G5 on PD	β_2_-microglobulin, urea, creatinine; dialysis clearance parameters	Modified Mini-Mental State Examination (3MS), RBANS, Trail Making Test A/B	Greater β_2_-microglobulin clearance was associated with better global cognition, executive function, and more favorable cognitive trajectory; Kt/V showed weaker associations	Clearance of middle molecules may reduce neurotoxic burden more effectively than removal of small water-soluble solutes
Murali et al. (2021) [27]	Longitudinal cohort; 211 CKD and ESKD patients	CKD stages 1–5 and dialysis	Overall uremic milieu	3MS, Trail Making Test A/B, Stroop, BDI-II	Dialysis initiation was associated with modest improvement in global cognition, particularly during the first year; executive deficits persisted in many patients	Partial reversibility of uremic brain dysfunction; persistent vascular injury, inflammation, and oxidative stress may sustain cognitive impairment
Brydges et al. (2021) [29]	Clinical cohort (PReDICT); 196 patients with major depressive disorder; non-CKD cohort; rs-fMRI subset *n* = 80	* Non-CKD population	IS and other indole metabolites	HAM-A, HAM-D, QIDS-SR; resting-state fMRI	Higher IS concentrations were associated with altered subcallosal cingulate connectivity and greater anxiety severity; functional connectivity partially mediated the association between IS and anxiety symptoms	Supports neuroactive properties of IS and potential effects on human brain networks independent of CKD

Abbreviations: 3MS, Modified Mini-Mental State Examination; AhR, aryl hydrocarbon receptor; BBB, blood–brain barrier; BDI-II, Beck Depression Inventory-II; BUN, blood urea nitrogen; CASI, Cognitive Abilities Screening Instrument; CES-D, Center for Epidemiologic Studies Depression Scale; CKD, chronic kidney disease; CKD-REIN, Chronic Kidney Disease–Renal Epidemiology and Information Network; CNS, central nervous system; ESKD, end-stage kidney disease; fMRI, functional magnetic resonance imaging; HAM-A, Hamilton Anxiety Rating Scale; HAM-D, Hamilton Depression Rating Scale; HD, hemodialysis; HDF, hemodiafiltration; HP, hemoperfusion; IAA, indole-3-acetic acid; iPTH, intact parathyroid hormone; IS, indoxyl sulfate; Hcy, homocysteine; Kt/V, dialysis urea clearance index; MMSE, Mini-Mental State Examination; MoCA, Montreal Cognitive Assessment; PAG, phenylacetylglutamine; PCS, p-cresyl sulfate; PD, peritoneal dialysis; PReDICT, Predictors of Remission in Depression to Individual and Combined Treatments; QIDS-SR, Quick Inventory of Depressive Symptomatology-Self-Report; RBANS, Repeatable Battery for the Assessment of Neuropsychological Status; rs-fMRI, resting-state functional magnetic resonance imaging; SPECT/CT, single-photon emission computed tomography/computed tomography; 99mTc-DTPA, technetium-99m diethylenetriaminepentaacetic acid; TMAO, trimethylamine N-oxide. * The only study in this Table that included non-CKD population.

### 4.2. Evidence from Animal Models

Experimental studies have substantially advanced understanding of the role of retained uremic solutes in central nervous system (CNS) injury, demonstrating that these metabolites represent active biological mediators rather than passive markers of declining kidney function. Animal models have shown that uremic toxins accumulate in the brain and disrupt essential processes involved in neuronal homeostasis, including inflammatory signaling, vascular integrity, synaptic regulation, and cellular stress responses (Table 2) [17,30,31,32,33]. Together with clinical observations, these findings support the concept that chronic exposure to the uremic milieu progressively reduces cerebral resilience across the spectrum of CKD-associated neurological dysfunction [12]. Progressive cognitive decline, impaired hippocampal synaptic plasticity, astrocytic activation, and hippocampal structural abnormalities have also been demonstrated after 5/6 nephrectomy, providing additional evidence that CKD can disrupt hippocampal function across behavioral, electrophysiological, and structural levels [34]. In the accompanying clinical component of the same study, patients with ESKD exhibited reduced hippocampal volume, providing translational support for the hippocampal abnormalities observed in the 5/6 nephrectomy model [34].

Among the retained solutes investigated, IS has been shown to promote oxidative stress and neuroinflammation by activating astrocytes and microglia, supporting its role in CKD-associated neurotoxicity [30]. Subsequent investigations have expanded these observations, demonstrating that IS influences multiple pathways involved in brain homeostasis, including microglial activation, astrocytic dysfunction, BBB impairment, altered synaptic regulation, and neuronal survival. Nevertheless, IS should be considered a representative component of a broader uremic environment rather than an isolated neurotoxin (Table 2).

Neuroinflammation represents one of the most consistent mechanisms linking uremic toxin accumulation with cognitive dysfunction. In a 5/6 nephrectomy mouse model, Li et al. (2021) demonstrated that cerebral accumulation of IS activated the NLRP3 inflammasome in microglia and astrocytes, resulting in increased production of pro-inflammatory cytokines, including IL-1β and IL-18, and subsequent impairment of hippocampal function [31]. Genetic deletion of NLRP3 attenuated cognitive deficits despite persistent renal dysfunction, whereas treatment with AST-120, which reduces multiple gut-derived uremic solutes, including IS, suppressed inflammasome activation and improved spatial learning and working memory [31]. These findings support a contribution of uremic toxin-driven inflammatory signaling to CKD-associated cognitive impairment, but do not establish IS as the principal causal mediator. Supporting this concept, Zimmermann et al. (2024) [32] reported that circulating uremic factors activate microglia through KCa3.1-mediated potassium efflux, promoting inflammasome activation and IL-1β release. Pharmacological inhibition of either KCa3.1 or IL-1 signaling improved cognitive performance, identifying microglial activation and downstream inflammatory pathways as potential therapeutic targets for uremic brain injury [32].

Retained solutes may also compromise neurovascular integrity. CKD models exhibit increased BBB permeability, facilitating brain exposure to circulating protein-bound metabolites, including IS, PCS, and IAA [17]. These effects were mediated partially by AhR activation, as AhR-deficient animals were substantially protected against CKD-induced BBB disruption and cognitive impairment. Complementary findings from Fang et al. (2023) demonstrated that serum from CKD mice, as well as combinations of gut-derived uremic toxins, impaired endothelial barrier function, increased cerebral permeability, promoted microhemorrhage formation, and activated microglia [33]. Together, these observations identify the BBB and neurovascular unit as critical interfaces through which systemic uremia influences the CNS.

Importantly, experimental interventions that reduce toxin accumulation provide further support for the contribution of retained solutes to CKD-associated neurological dysfunction. AST-120 treatment attenuated inflammatory activation, improved cognitive performance, and partially restored cerebral homeostasis in CKD models [35]. Because AST-120 is not selective for IS, these effects are more appropriately interpreted as evidence for a potential benefit of reducing the overall uremic toxin burden rather than proof of an IS-specific causal effect.

Animal studies therefore support a multifactorial model in which retained uremic solutes initiate interconnected neurovascular, inflammatory, metabolic, and synaptic abnormalities. IS is best regarded as a representative component of this environment rather than as an isolated explanation for the neurological complications of CKD.

**Table 2:** In vivo experimental evidence for CKD-associated brain dysfunction and uremic neurotoxicity.

Study	Experimental Model and Exposure	Uremic Factor(s) Studied	Outcomes Assessed	Key Findings	Mechanistic Interpretation
Adesso et al. (2017) [30]—in vivo component of the study	Mice receiving acute intraperitoneal IS (800 mg/kg)	IS	Brain histology; COX-2 expression; nitrotyrosine formation	IS exposure induced histological brain alterations and increased COX-2 expression and nitrotyrosine formation.	Direct IS exposure promotes cerebral inflammatory and oxidative/nitrosative injury.
Watanabe et al. (2021) [36]	Rats with adenine-induced CKD (0.5% adenine diet)	Uremic milieu	Morris Water Maze; hippocampal histology; oxidative-stress markers	CKD was associated with hippocampal neuronal pyknosis and increased oxidative stress, despite limited behavioral impairment.	Oxidative stress may produce hippocampal injury before consistent behavioral deficits become detectable.
Bobot et al. (2020) [17]	Adenine-fed rats, 5/6 nephrectomy rats, and AhR-knockout mice; AST-120 intervention	IS, PCS and IAA	Novel Object Recognition, Object Location, and Social Memory tests; BBB permeability; AhR-related analyses	CKD increased BBB permeability and impaired memory. AhR deficiency was protective, whereas AST-120 reduced uremic toxin burden, including IS, but incompletely restored BBB integrity.	AhR activation contributes to BBB disruption, increased cerebral exposure to circulating solutes, and cognitive dysfunction.
Li et al. (2021) [31]	5/6 nephrectomy mice and NLRP3-knockout mice; AST-120 intervention	IS and PCS	Y-maze; Morris Water Maze; cerebral toxin accumulation; inflammasome and cytokine analyses	CKD impaired working memory and spatial learning. AST-120 and NLRP3 deletion attenuated cognitive deficits and inflammatory activation.	Accumulated uremic toxins activate inflammasome-related signaling in microglia and astrocytes, increasing IL-1β and IL-18 and impairing hippocampal function.
Yu et al. (2022) [34]	Rats subjected to 5/6 nephrectomy	Uremic milieu	Novel Object Recognition, Y-maze, Barnes maze, and fear conditioning; hippocampal LTP; astrocytic and BBB-related markers	Progressive CKD produced cognitive decline, reduced hippocampal LTP, astrocytic activation, and hippocampal dysfunction.	Gliosis, altered AQP4 and NHE-1 signaling, BBB dysfunction, and impaired synaptic plasticity may jointly contribute to cognitive impairment.
Sun et al. (2020) [37]	Unilateral-nephrectomy mice receiving PCS; AST-120 intervention	PCS	Morris Water Maze, Open Field, Forced Swim, Tail Suspension, and Light/Dark Box tests; neurogenesis, apoptosis, and BDNF/TrkB analyses	PCS induced learning and memory deficits and anxiety- and depression-like behavior; AST-120 attenuated these abnormalities.	Oxidative stress, NF-κB activation, impaired neurogenesis, apoptosis, reduced BDNF/TrkB signaling, and neurotransmitter dysfunction contribute to PCS neurotoxicity.
Fang et al. (2023) [33]	Aged mice with adenine-induced CKD	Uremic milieu	BBB leakage; cerebral microhemorrhages; endothelial integrity; microglial activation	CKD increased BBB permeability, cerebral microhemorrhage formation, and microglial activation.	Endothelial and BBB dysfunction translate systemic uremia into cerebral vascular injury and neuroinflammation.
Yu et al. (2024) [35]	5/6 nephrectomy rats treated with AST-120	Predominantly IS within the uremic milieu	Novel Object Recognition, Y-maze, Barnes maze; hippocampal oscillations and LTP; GFAP, AQP4, and BBB-related analyses	AST-120 reduced the uremic toxin burden, including IS, and improved cognition, hippocampal oscillations, LTP, and behavioral performance.	Reducing toxin burden may limit astrocytic activation, AQP4-associated BBB dysfunction, neuroinflammation, and impaired synaptic plasticity.
Karbowska et al. (2020) [38]	Wistar rats receiving chronic IS administration	IS	T-maze, Open Field, Splash, and Elevated Plus Maze tests; brain IS concentrations; monoamines and lipid peroxidation	Chronic IS exposure caused dose-dependent learning, memory, and behavioral abnormalities with regional brain accumulation and altered monoamine concentrations.	Brain accumulation of IS promotes oxidative stress and monoaminergic dysregulation.
Sun et al. (2021) [39]	Unilateral-nephrectomy mice receiving IS; AST-120 intervention	IS	Morris Water Maze, Open Field, Forced Swim, and Tail Suspension tests; serum, CSF, and brain IS; neurogenesis, neuronal survival, BDNF/TrkB, AhR, and inflammatory markers	IS accumulated in serum, CSF, and brain and induced cognitive, anxiety-like, and depression-like abnormalities; AST-120 ameliorated these effects.	Oxidative stress, neuroinflammation, impaired neurogenesis, neuronal loss, reduced BDNF/TrkB signaling, and AhR activation contribute to IS-induced brain dysfunction.
Zimmermann et al. (2024) [32]	Mice subjected to 5/6 nephrectomy and treated with TRAM-34 or anakinra	Circulating uremic factors	Non-Spatial Object Recognition and Radial Arm Maze; BBB integrity; microglial potassium handling; inflammasome and IL-1β signaling	CKD caused BBB disruption, microglial activation, and cognitive impairment; KCa3.1 inhibition and IL-1 receptor blockade improved cognition.	KCa3.1-mediated potassium efflux and downstream inflammasome/IL-1β signaling link circulating uremic factors to microglial activation and cognitive dysfunction.
Mazumder et al. (2019) [40]	Mice with adenine-induced CKD	Uremic milieu	Object Recognition, Object Location Memory, and Forced Swim tests; mitochondrial respiratory-chain activity; oxidative, cholinergic, astrocytic, and dendritic markers	CKD caused cognitive impairment, depression-like behavior, hippocampal injury, mitochondrial dysfunction, astrocytic activation, and dendritic spine loss.	Mitochondrial bioenergetic failure, oxidative stress, cholinergic dysfunction, gliosis, and synaptic structural injury act as convergent mechanisms.

Abbreviations: AhR, aryl hydrocarbon receptor; Akt, protein kinase B; AQP4, aquaporin 4; AST-120, oral carbon adsorbent; BBB, blood–brain barrier; BDNF, brain-derived neurotrophic factor; CKD, chronic kidney disease; COX-2, cyclooxygenase-2; CSF, cerebrospinal fluid; GFAP, glial fibrillary acidic protein; IAA, indole-3-acetic acid; IL, interleukin; IS, indoxyl sulfate; KCa3.1, intermediate-conductance calcium-activated potassium channel 3.1; LTP, long-term potentiation; NF-κB, nuclear factor κB; NHE-1, sodium–hydrogen exchanger isoform 1; NLRP3, NLR family pyrin domain containing 3; PCS, p-cresyl sulfate; PDK1, 3-phosphoinositide-dependent protein kinase 1; PI3K, phosphoinositide 3-kinase; ROS, reactive oxygen species; TRAM-34, 1-[(2-chlorophenyl)diphenylmethyl]-1H-pyrazole; TrkB, tropomyosin receptor kinase B.

### 4.3. In Vitro and Ex Vivo Models of Uremic Neurotoxicity

Cellular studies provide important mechanistic evidence that retained uremic solutes directly affect components of the CNS and neurovascular unit, including neurons, astrocytes, microglia, and brain endothelial cells (Table 3) [35,41,42,43]. Although these models cannot fully reproduce the complexity of the human uremic environment, they enable investigation of toxin-specific effects under controlled conditions.

Across diverse in vitro models, the protein-bound uremic toxin IS has consistently exhibited pronounced neurotoxic effects. Using hippocampal neuronal cells, Watanabe et al. (2021) showed that IS and related retained metabolites reduced neuronal viability in a concentration-dependent manner [36]. Moreover, combined exposure to multiple metabolites produced greater cytotoxic effects than exposure to individual compounds, supporting the concept that CKD-associated neurological injury results from cumulative effects of the uremic milieu rather than a single dominant toxin [36].

Astrocytes represent another important cellular target of uremic neurotoxicity because of their roles in neuronal metabolic support, synaptic maintenance, and neurovascular regulation. Lin et al. (2019) demonstrated that clinically relevant concentrations of IS induced apoptosis in human astrocytes through oxidative injury and mitochondrial membrane dysfunction [41]. Jeong et al. (2023) further showed that IS exposure impaired astrocytic metabolic pathways, reducing glycolytic activity and increasing susceptibility to cellular stress [42].

Beyond direct cellular toxicity, uremic toxins promote inflammatory activation within the neurovascular unit. Adesso et al. (2018) demonstrated that IS induced glial activation characterized by increased production of inflammatory mediators and oxidative stress markers, mediated partly through NF-κB and AhR signaling pathways [43]. Importantly, conditioned medium from IS-treated glial cells induced secondary neuronal injury, indicating that activated glia may amplify rather than solely reflect toxin-mediated brain injury.

Taken together, cellular studies indicate that uremic toxins influence multiple components of the CNS through interconnected pathways involving inflammatory activation, metabolic disturbance, and impaired cellular communication. These findings complement clinical and animal data and support a model in which chronic exposure to the uremic milieu progressively reduces neuronal resilience.

**Table 3:** In vitro and ex vivo evidence for uremic toxin-mediated neurotoxicity in CKD.

Author (Year)	Experimental Model	Uremic Toxin(s)	Methods/Exposure	Main Findings	Proposed Mechanism(s)
Adesso et al. (2018) [43]	C6 astrocytes, primary astrocytes, primary microglia, mixed glial cultures, cortical and hippocampal neurons; human CKD serum	IS	Cells exposed to IS (15–60 μM) or CKD serum; ROS, NO, cytokines, NF-κB/AhR activation, neuronal viability assessed; AST-120 used to adsorb IS	IS induced marked glial activation with increased ROS, nitric oxide, TNF-α, IL-6, nitrotyrosine, and neuronal death. AST-120 attenuated inflammatory responses and neurotoxicity.	IS activates NF-κB and AhR, promotes oxidative stress and neuroinflammation, decreases HO-1 expression, and induces indirect neuronal injury through activated glia.
Watanabe et al. (2021) [36]	HT-22 mouse hippocampal neuronal cells	IS, IAA, indole, methylglyoxal, PCS, hippurate, creatinine, methylguanidine, guanidinosuccinate	Cells exposed to individual toxins (100–1000 μM) or toxin mixtures; MTT assay, glutathione measurement, Western blot, qPCR	IS, methylglyoxal, indole, and IAA reduced neuronal viability in a concentration-dependent manner. Combined toxin exposure produced greater cytotoxicity than individual toxins.	Oxidative stress (↓ glutathione), activation of NRF2, increased p47phox and IL-6, suggesting synergistic neurotoxicity of the uremic milieu.
Lin et al. (2019) [41]	Primary human astrocytes	IS	IS (1–20 μM); viability assays, ROS measurement, mitochondrial membrane potential, RNA sequencing, Western blot	IS induced dose- and time-dependent astrocyte apoptosis, increased ROS production, mitochondrial dysfunction, and widespread transcriptional changes.	Oxidative stress, mitochondrial injury, activation of NRF2, upregulation of DUSP1/5/8/16, suppression of MAPK (ERK/JNK/p38) signaling, leading to apoptosis.
Jeong et al. (2023) [42]	Primary human astrocytes	IS	IS (0.5–1.0 mM); MTT assay, flow cytometry, extracellular acidification rate (ECAR), Western blot	IS reduced astrocyte viability and induced apoptosis while markedly suppressing glycolysis.	Metabolic dysfunction through downregulation of glycolytic enzymes (HK1, PFKP, PDH, PKM2, LDHA), resulting in energy failure and apoptotic cell death.
Natale et al. (2021) [44]	Rat hippocampal slices (electrophysiology); exposure to dialysis patient serum	Guanidine compounds and uremic serum	Bath application of guanidine (1 μM–1 mM) or dialysis serum; field EPSP recordings and AMPA/NMDA current analysis	Guanidine compounds and uremic serum increased glutamatergic transmission, enhancing AMPA/NMDA receptor-mediated responses and presynaptic glutamate release.	Synaptic hyperexcitability, enhanced glutamatergic neurotransmission, excitotoxicity, and potential neurodegeneration.

Abbreviations: AhR, aryl hydrocarbon receptor; AMPA, α-amino-3-hydroxy-5-methyl-4-isoxazolepropionic acid receptor; AST-120, spherical carbon adsorbent; CKD, chronic kidney disease; DUSP, dual-specificity phosphatase; ECAR, extracellular acidification rate; EPSP, excitatory postsynaptic potential; ERK, extracellular signal-regulated kinase; HK1, hexokinase 1; HO-1, heme oxygenase-1; HT-22, mouse hippocampal neuronal cell line; IAA, indole-3-acetic acid; IL, interleukin; IS, indoxyl sulfate; JNK, c-Jun N-terminal kinase; LDHA, lactate dehydrogenase A; MAPK, mitogen-activated protein kinase; MTT, 3-(4,5-dimethylthiazol-2-yl)-2,5-diphenyltetrazolium bromide; NF-κB, nuclear factor κB; NMDA, N-methyl-D-aspartate receptor; NO, nitric oxide; NRF2, nuclear factor erythroid 2-related factor 2; p38 MAPK, p38 mitogen-activated protein kinase; p47phox, 47-kDa phagocyte oxidase subunit; PCS, p-cresyl sulfate; PDH, pyruvate dehydrogenase; PFKP, phosphofructokinase, platelet type; PKM2, pyruvate kinase M2; qPCR, quantitative polymerase chain reaction; ROS, reactive oxygen species; TNF-α, tumor necrosis factor-α.

## 5. Cerebrovascular and Neurovascular Dysfunction

Cerebrovascular dysfunction is a major contributor to chronic cognitive impairment in CKD and may also reduce tolerance to acute metabolic or osmotic stress (Table 4). Because the kidney and brain share low-resistance microvascular networks exposed to high-volume blood flow, both are vulnerable to endothelial injury, hemodynamic stress, and maladaptive vascular remodeling. CKD may therefore be viewed partly as a systemic small-vessel disorder in which cerebral microvascular injury reduces structural and functional brain reserves [20,45].

### 5.1. Cerebral Small-Vessel Disease in CKD

Cerebral small-vessel disease (CSVD) is the most consistently reported cerebrovascular manifestation of CKD. Neuroimaging studies show increased white matter hyperintensities, lacunes, cerebral microbleeds, enlarged perivascular spaces, and brain atrophy, particularly in advanced CKD and dialysis populations; these abnormalities correlate with cognitive decline [20,45]. Consistent with this vascular pattern, early cognitive deficits predominantly affect attention, executive function, orientation, language, and praxis, while episodic memory may be relatively preserved [46].

Large-scale imaging data further support the association between renal dysfunction and cerebral microvascular injury. In the UK Biobank, lower creatinine- and cystatin C-based eGFR and higher blood urea nitrogen were associated with greater white matter hyperintensity volume, impaired white matter microstructure, lacunes, enlarged perivascular spaces, microbleeds, and higher overall CSVD burden. Mendelian randomization also linked genetically predicted higher eGFR to lower white matter hyperintensity burden, supporting a potential causal contribution of renal dysfunction to CSVD [47].

Multicenter cohorts of patients with acute ischemic stroke or intracerebral hemorrhage similarly show that declining kidney function independently predicts greater CSVD and cerebrovascular injury [48,49]. CKD is also associated with cerebral microbleeds and cortical atrophy beyond the effects of aging and hypertension [50]. In the Framingham Heart Study, albuminuria rather than reduced eGFR independently predicted CSVD and subsequent vascular dementia and Alzheimer’s disease, further implicating systemic microvascular injury in the kidney–brain axis [51].

#### CKD-Specific Mechanisms of Cerebrovascular Dysfunction

Beyond shared vascular risk factors, CKD may exert disease-specific effects on the cerebral microcirculation. Endothelial dysfunction, inflammation, oxidative stress, retained uremic solutes, mineral-metabolism disturbances, arterial stiffness, and blood pressure variability interact to impair vascular compliance and cerebral perfusion, progressively reducing cerebrovascular reserves [14,45,52].

Endothelial dysfunction appears to be an important contributor to CKD-related vascular injury. Experimental evidence indicates that renal failure reduces endothelial nitric oxide production through impaired endothelial nitric oxide synthase (eNOS) phosphorylation, thereby compromising endothelium-dependent vasorelaxation [53]. At the cerebral level, patients receiving hemodialysis exhibit attenuated cerebrovascular reactivity to carbon dioxide, consistent with diminished vasodilatory reserves and a reduced capacity of the cerebral circulation to adapt blood flow to changing physiological demands [54]. Experimental work has also shown that CKD-related sympathetic overactivity impairs endothelium-derived hyperpolarization by downregulating connexin-43 at myoendothelial junctions, providing a mechanistic link between autonomic dysregulation and defective neurovascular signaling [55]. Consistent with widespread endothelial injury, patients with advanced CKD exhibit elevated circulating concentrations of soluble vascular cell adhesion molecule-1 (sVCAM-1) and thrombomodulin even before the initiation of kidney replacement therapy, reflecting sustained endothelial activation and loss of vascular homeostasis [56].

Arterial stiffening may further intensify cerebral microvascular injury. Medial vascular calcification associated with disturbances in phosphate, calcium, fibroblast growth factor-23, and parathyroid hormone metabolism reduces arterial compliance and increases transmission of pulsatile energy to cerebral microvessels. The resulting mechanical stress can promote endothelial and BBB injury, CSVD, and impaired buffering of systemic blood pressure fluctuations [14,45,52].

Other systemic factors may amplify this process. In peritoneal dialysis, protein-energy wasting, reflected by a lower normalized protein catabolic rate, has been independently associated with lacunar infarction, white matter hyperintensities, and poorer cognitive performance, suggesting an additional contribution of malnutrition to vascular and inflammatory injury [57].

Thus, CSVD in CKD likely reflects cumulative endothelial, arterial, autonomic, mineral-metabolic, and systemic injury rather than a coincidental comorbidity. This structural vulnerability contributes to chronic cognitive impairment and may reduce tolerance to acute uremic, inflammatory, electrolyte, or hemodynamic stress.

### 5.2. Impaired Neurovascular Coupling

In addition to structural cerebrovascular injury, impaired neurovascular coupling (NVC), the dynamic matching of regional neuronal activity and cerebral blood flow, has emerged as an important functional abnormality in advanced CKD and ESKD [58]. NVC depends on coordinated signaling among neurons, astrocytes, endothelial cells, pericytes, and vascular smooth muscle cells; disruption of this network may impair adjustment of regional perfusion to metabolic demand [59].

Resting-state fMRI and arterial spin-labeling studies have identified global and regional mismatches between cerebral perfusion and spontaneous neuronal activity in advanced CKD and ESKD. Abnormal coupling has been reported across frontal, cingulate, parietal, temporal, limbic, precuneus, and default-mode regions, although its distribution varies among studies. These abnormalities are associated with poorer global cognition and deficits in memory, attention, and executive function [58,60,61,62,63,64].

Cross-sectional data suggest that NVC abnormalities become more pronounced with advanced CKD and coexist with altered cerebral blood flow, spontaneous neuronal activity, and network integration. Associations with dialysis adequacy, serum urea, anemia, pulse pressure, and phosphorus suggest that metabolic and hemodynamic disturbances jointly contribute to impaired cerebrovascular regulation [58,61,63,64].

Experimental findings provide a plausible mechanistic basis for the NVC abnormalities observed in advanced CKD. Impaired endothelial signaling and sympathetic dysregulation may disturb the regulation of vascular tone and communication between endothelial and vascular smooth muscle cells [55]. In parallel, oxidative stress, chronic inflammation, and exposure to retained uremic solutes may compromise endothelial barrier function and the integrity of the broader neurovascular unit [17,33]. Dysfunction of other neurovascular components, including pericytes and astrocytes, may further impair the coordination between neuronal activity and regional cerebral perfusion [59]. Collectively, these alterations may reduce the capacity of the cerebral circulation to respond adequately to changing metabolic demands, thereby increasing susceptibility to persistent cognitive dysfunction and acute neurological deterioration during severe uremia or hemodynamic stress.

NVC dysfunction may be partly reversible. Twenty-four hours after a hemodialysis session, NVC and several cognitive measures improved but remained below control levels. Greater NVC improvement was associated with higher hemoglobin, lower homocysteine, smaller ultrafiltration volumes, more stable intradialytic systolic blood pressure, and better immediate and long-term memory [64]. These findings suggest that NVC reflects both chronic microvascular injury and dynamic, potentially modifiable effects of uremic and metabolic disturbances.

Impaired NVC may therefore contribute to cognitive dysfunction while limiting cerebral tolerance to severe metabolic, inflammatory, or hemodynamic stress in advanced kidney failure.

### 5.3. Cerebral Autoregulation and Hemodialysis-Related Hypoperfusion

NVC and cerebral autoregulation are complementary mechanisms of cerebrovascular stability. NVC matches regional blood flow to neuronal activity, whereas autoregulation stabilizes cerebral perfusion across changes in systemic blood pressure [58,59,65]. Dysfunction of either mechanism, particularly when combined, may reduce cerebrovascular reserves [66,67].

Evidence for impaired autoregulation in non-dialysis CKD is heterogeneous, with preserved dynamic autoregulation reported in some stage 3–4 cohorts. Abnormalities are more consistently observed in advanced kidney failure and during hemodialysis, where endothelial dysfunction, arterial stiffness, vascular calcification, chronic hypertension, and impaired vascular reactivity may limit the ability of the cerebral circulation to buffer systemic hemodynamic fluctuations [66,67].

Clinical data support the consequences of reduced vascular adaptability. In older patients with stage 4–5 CKD, greater aortic stiffness was associated with poorer cognition, although only psychomotor speed remained significant after adjustment and no independent association with structural MRI abnormalities was found [68]. In maintenance hemodialysis, a previous stroke and lower diastolic blood pressure were independently associated with poorer global cognition [69].

Hemodialysis imposes additional hemodynamic stress through rapid volume shifts, ultrafiltration, reduced cardiac output and blood pressure, and limited vascular reactivity. Cerebral oximetry identified transient ischemic events, defined by a >15% relative fall in cerebral oxygen saturation, during approximately one-quarter of sessions; larger reductions in mean arterial pressure were associated with more events, although no universally safe pressure threshold could be identified [70].

Using oxygen-15-labeled water positron emission tomography ([15O]H2O PET), Polinder-Bos et al. demonstrated an acute decline in global and regional cerebral blood flow during conventional hemodialysis; a higher ultrafiltration volume and rate were associated with lower cerebral blood flow [71].

Impaired cerebral autoregulation was reported in 37.3% of monitored hemodialysis sessions. Diabetes mellitus and an intradialytic systolic blood pressure fall of >20 mmHg were associated with poorer autoregulatory function, linking both pre-existing vascular disease and treatment-related stress to perfusion instability [72].

Regional cerebrovascular reactivity may further modify the response to hemodialysis. Lower reactivity in the anterior cingulate, insular cortex, and posterior parahippocampal gyrus has been associated with greater intradialytic cerebral oxygen desaturation [73], which longitudinal observations link to subsequent cognitive deterioration.

Repeated dialysis-related hypoperfusion may therefore produce cumulative cerebral injury. Recurrent hemodynamic stress has been linked to white matter injury, loss of structural integrity, and cognitive decline, although effects vary substantially among patients [74]. Rather than directly causing UE, impaired autoregulation and recurrent hypoperfusion may reduce cerebral resilience to severe azotemia, electrolyte disturbances, inflammation, or osmotic shifts.

Accordingly, hemodialysis-related perfusion instability may act as a dynamic amplifier of pre-existing cerebrovascular vulnerability.

### 5.4. Blood–Brain Barrier and Neurovascular Unit Dysfunction

The BBB is an integral component of the neurovascular unit, whose integrity depends on coordinated endothelial, pericytic, astrocytic, neuronal, and immune interactions. CKD-associated endothelial activation, oxidative stress, inflammation, and retained solutes can destabilize this system, making BBB dysfunction an active mechanism of cerebral injury [75].

Experimental CKD is associated with reduced claudin-5, occludin, and zonula occludens-1, basement-membrane disruption, increased permeability, and cerebral microhemorrhages [33,76,77,78,79]. Protein-bound toxins including IS, PCS, IAA, and trimethylamine N-oxide can impair barrier function [80], while early pericyte dysfunction may further destabilize the neurovascular unit [81].

BBB disruption may amplify the interaction between systemic and cerebral inflammation by facilitating entry of inflammatory mediators and retained solutes, while circulating cytokines can further impair endothelial integrity [79,82,83]. Retained toxins can additionally activate inflammatory signaling in brain endothelial cells and interfere with CNS solute-clearance transporters [78,84,85,86,87].

Experimental models suggest that BBB dysfunction can occur early. Urea-mediated MMP-2 activation increases permeability, with insoluble tau accumulation before overt neurodegeneration [77]. Adenine-induced CKD likewise produces marked BBB disruption despite minimal neuronal loss [88].

Human evidence remains limited and relies substantially on circulating biomarkers and peripheral surrogate models. In a cross-sectional comparison, patients receiving maintenance hemodialysis had altered circulating neuronal markers and reduced tight-junction protein expression in subcutaneous microvessels [89]. These findings support systemic microvascular barrier dysfunction in the uremic environment. However, because the tissue experiments used subcutaneous rather than cerebral microvessels, they do not provide direct evidence of human BBB disruption.

Complementary longitudinal findings indicate that restoration of kidney function may modify circulating markers of neuronal and endothelial stress. After kidney transplantation, changes in circulating neuronal, endothelial, and inflammatory markers suggested modification of neurovascular stress [90]. These observations suggest that correction of the uremic milieu may influence neurovascular and endothelial homeostasis, although the study did not directly assess BBB permeability or tight-junction integrity.

Preliminary human evidence was provided by Bobot et al. (2024), who directly assessed BBB permeability in 15 patients with ESKD and 15 healthy controls [18]. Patients with ESKD showed increased BBB permeability together with higher protein-bound uremic toxin concentrations, enhanced AhR-activating potential, and poorer cognitive performance [18]. Although the direct assessment of BBB permeability is an important strength of the study, the small sample size limits the strength of the conclusions, which require confirmation in larger cohorts. Overall, human studies support BBB involvement in advanced kidney disease but differ in measurement directness, underscoring the need for studies combining biomarkers with quantitative BBB imaging.

**Table 4:** Cerebrovascular and neurovascular mechanisms contributing to chronic cognitive impairment and susceptibility to acute encephalopathic decompensation in CKD.

Cerebrovascular or Neurovascular Mechanism	Contribution to Chronic Cognitive Impairment	Potential Contribution to Acute Encephalopathic Decompensation	Principal Pathophysiological Links
Endothelial dysfunction [45,52,53,54,56]	Impaired endothelial vasoregulation and sustained endothelial activation may promote cerebral hypoperfusion, neurovascular dysfunction, and small-vessel injury.	Reduced cerebrovascular reserves may increase vulnerability to systemic hypotension, severe metabolic disturbances, and other hemodynamic stressors.	Uremic toxins; inflammation; oxidative stress; reduced nitric oxide bioavailability; impaired cerebrovascular reactivity.
Blood–brain barrier and pericyte dysfunction [18,33,76,77,80,81]	Increased cerebral exposure to plasma proteins, retained solutes, and inflammatory mediators may promote glial activation, white matter injury, and persistent neuronal dysfunction.	Increased cerebral exposure to retained solutes and inflammatory mediators may amplify cerebral edema, neuronal excitability, and metabolic dysfunction during severe uremia or systemic inflammation.	Tight-junction disruption; matrix metalloproteinase activation; endothelial injury; pericyte dysfunction; loss of neurovascular unit integrity.
Cerebral small-vessel disease [20,47,48,49,50,51]	White matter hyperintensities, lacunes, cerebral microbleeds, enlarged perivascular spaces, and brain atrophy contribute particularly to executive dysfunction and reduced processing speed.	Reduced structural brain reserves may increase the severity of neurological manifestations during acute metabolic or hemodynamic disturbances.	Hypertension; arterial stiffness; endothelial dysfunction; albuminuria; inflammation; CKD-specific microvascular injury.
Impaired neurovascular coupling [58,59,60,61,62,63]	A mismatch between neuronal activity and regional cerebral perfusion may contribute to cognitive slowing and deficits in memory, attention, and executive function.	Limited dynamic perfusion responses may increase vulnerability to acute cerebral metabolic insufficiency during severe systemic or uremic stress.	Disrupted coordinated signaling among neurons, astrocytes, endothelial cells, pericytes, and vascular smooth muscle cells within the neurovascular unit.
Impaired cerebral autoregulation [66,67,72]	Pressure-dependent fluctuations in cerebral perfusion may contribute to recurrent ischemic stress and cumulative brain injury, particularly in advanced kidney failure and during hemodialysis.	Failure to stabilize cerebral blood flow during systemic or intradialytic hypotension may precipitate acute neurological deterioration.	Arterial stiffness; chronic hypertension; autonomic dysregulation; endothelial dysfunction; limited cerebrovascular reactivity.
Hemodialysis-related cerebral hypoperfusion [70,71,72,73]	Recurrent reductions in cerebral perfusion and oxygenation may contribute to cumulative white matter injury, loss of cerebral structural integrity, and cognitive decline.	Acute reductions in cerebral perfusion and oxygenation may worsen neurological dysfunction in patients with limited cerebrovascular reserve.	Ultrafiltration; intradialytic hypotension; reduced cardiac output; impaired cerebrovascular reactivity; limited autoregulatory capacity.
Arterial stiffness and vascular calcification [14,45,52,68]	Transmission of excessive pulsatile energy may damage cerebral microvessels, while greater arterial stiffness has been linked to poorer psychomotor performance.	Reduced vascular compliance may limit adaptation to abrupt changes in systemic blood pressure and cerebral perfusion.	CKD-mineral and bone disorder; altered phosphate and calcium metabolism; fibroblast growth factor 23 and parathyroid hormone dysregulation; inflammation; oxidative stress; extracellular-matrix remodeling.

Abbreviations: CKD, chronic kidney disease. Note: The proposed contributions to acute encephalopathic decompensation are based primarily on mechanistic and associative evidence and should not be interpreted as evidence of an obligatory or linear progression from chronic cognitive impairment to uremic encephalopathy.

## 6. Systemic Inflammation and Neuroinflammation

### 6.1. Systemic Inflammatory Activation in CKD

CKD is characterized by persistent low-grade systemic inflammation, which may arise early in the course of renal dysfunction and become more pronounced as kidney function declines. Chronic inflammation is therefore regarded as an integral component of CKD pathophysiology, contributing to disease progression and injury in multiple extra-renal organs, including the CNS. The inflammatory milieu arises from the combined effects of uremic toxin retention, oxidative stress, metabolic acidosis, intestinal dysbiosis, recurrent infections, dialysis-related bioincompatibility, and persistent renal tissue injury. Together, these factors create a self-perpetuating cycle in which inflammation both results from and further promotes kidney damage [91,92,93,94].

Pro-inflammatory mediators including TNF-α, IL-1β, IL-6, and C-reactive protein (CRP) are frequently elevated in CKD. Beyond systemic complications, persistent cytokine signaling can impair endothelial function, promote vascular inflammation, and facilitate peripheral immune-to-brain communication, providing a plausible link to cerebral dysfunction [82,83,91].

Biochemical evidence of chronic systemic inflammation has been reported in approximately 30–50% of patients with advanced CKD, including those receiving maintenance hemodialysis or peritoneal dialysis [95,96]. Persistent elevation of inflammatory biomarkers, particularly CRP and IL-6, is associated with greater cardiovascular morbidity and independently predicts adverse outcomes and mortality in dialysis populations [96,97]. Chronic inflammation may also contribute to protein-energy wasting, frailty, and the premature-aging phenotype observed in advanced CKD. Regardless, its independent contribution to cognitive and functional decline remains incompletely established [92].

Composite indices such as the systemic immune-inflammation index may predict CKD progression and adverse outcomes, but they remain markers of inflammatory activity rather than evidence of a direct causal role in neurological complications [94,98].

Systemic inflammation is closely intertwined with widespread endothelial dysfunction, another major feature of CKD that extends beyond the renal vasculature. Multiple disease-related factors, including oxidative stress, asymmetric dimethylarginine accumulation, hyperhomocysteinemia, insulin resistance, calcium-phosphate imbalance, premature vascular aging, and chronic immune activation, may act synergistically to reduce endothelial nitric oxide bioavailability, disrupt vascular homeostasis, and promote a prothrombotic and pro-inflammatory endothelial phenotype [99,100,101,102,103,104]. At the same time, intrarenal inflammatory pathways driven by infiltrating macrophages and resident immune cells promote tubulointerstitial fibrosis and nephron loss, further amplifying systemic inflammatory signaling and reinforcing the cycle between progressive renal injury and chronic immune activation [105,106,107,108].

At the brain interface, endothelial dysfunction and persistent immune activation may compromise neurovascular homeostasis, promote BBB disruption, and facilitate neuroglial activation, linking systemic inflammation to CKD-associated brain dysfunction.

### 6.2. Microglial Activation and Inflammatory Signaling

BBB and neurovascular-unit disruption may expose the brain to circulating cytokines, inflammatory mediators, and retained uremic solutes, promoting coordinated activation of microglia and astrocytes [18,33]. In advanced CKD, this neuroimmune response may affect synaptic transmission, neuronal metabolism, and cerebral homeostasis [109].

Microglia are central mediators of this response. Although homeostatic microglia support neuronal and synaptic function [110], circulating uremic factors in CKD can induce sustained activation, altered ion handling, inflammatory signaling, and increased IL-1β production, thereby contributing to neuronal dysfunction [32,111].

Experimental evidence suggests that CKD plasma may promote KCa3.1-dependent potassium efflux and p38 MAPK signaling in microglia, with subsequent increases in pro-IL-1β expression [32]. Cell-specific experiments further indicate that microglial IL-1β maturation under CKD conditions can occur independently of canonical NLRP3–caspase-1 signaling [111].

One proposed non-canonical mechanism may involve the lysosomal CTSC–caspase-8 signaling pathway. In experimental CKD, microglia exhibit increased expression of inflammatory and lysosomal genes, including CTSC and caspase-8. In vitro, exposure to CKD plasma increases CTSC activity, caspase-8 activation, and IL-1β maturation, whereas CTSC inhibition or knockdown attenuates these responses and reduces markers of microglial activation [111]. Microglia-derived IL-1β acts on neuronal interleukin-1 receptor type 1 (IL-1R1), disrupting neuronal potassium homeostasis and interfering with synaptic plasticity signaling, which ultimately contributes to cognitive dysfunction. Pharmacological inhibition of IL-1R signaling ameliorates these abnormalities in experimental CKD, supporting this neuroimmune pathway as a potential therapeutic target [32].

In addition to cytokine production, activated microglia undergo substantial functional reprogramming. Experimental CKD is associated with increased Iba1 immunoreactivity and intracellular CD68 burden, together with an accumulation of TMEM119-positive resident microglia. These changes are accompanied by enhanced lysosomal activity and preferential engulfment of excitatory synaptic components. Persistent microglial activation may therefore promote excessive synapse elimination, reduced excitatory synaptic density, impaired hippocampal long-term potentiation, and cognitive dysfunction. The CCL4–CCR5 signaling axis may contribute to this process by promoting microglial recognition and engulfment of excitatory synapses, whereas CCR5 knockdown reduces synaptic phagocytosis and improves synaptic and cognitive function in experimental CKD [112].

CKD may also impair the resolution of neuroinflammation after a secondary cerebral insult. In adenine-induced CKD mice subjected to ischemic stroke, Kaur et al. (2025) [113] identified defective microglial efferocytosis related to ADAM17-mediated MerTK cleavage, enhanced neuroinflammatory activation, and poorer long-term sensorimotor recovery. Desidustat improved efferocytosis and functional outcome, suggesting that impaired clearance of apoptotic cells and ADAM17–MerTK signaling may contribute to prolonged cerebral inflammation and injury in this experimental setting [113].

Accumulating evidence also indicates that uremic toxins can participate directly in neuroglial activation, in addition to their effects on systemic inflammatory pathways. Exposure of astrocytes and mixed glial cultures to IS increases ROS production, suppresses nuclear factor erythroid 2-related factor 2 (NRF2)-dependent antioxidant defenses, and activates aryl hydrocarbon receptor (AhR) and NF-κB signaling. These responses are accompanied by increased expression or release of TNF-α, IL-6, iNOS, and COX-2 [30]. These findings support the view that IS is not only a retained metabolic waste product but a biologically active mediator capable of promoting neuroimmune dysfunction within the kidney–brain axis.

### 6.3. Reactive Astrogliosis and Glial Crosstalk

Astrocytes provide metabolic and trophic support, regulate glutamate and extracellular ions, maintain synaptic homeostasis, and contribute to BBB function [114,115]. Persistent inflammatory stimulation can induce reactive states characterized by impaired glutamate clearance, altered energy metabolism, reduced neuronal support, and impaired neurovascular homeostasis.

Microglia and astrocytes communicate bidirectionally through cytokines, chemokines, ROS, purinergic signals, and other mediators. This reciprocal signaling can sustain neuroglial inflammation beyond the initiating stimulus [115,116].

Prolonged inflammatory and oxidative signaling may impair synaptic transmission, mitochondrial function, glutamate handling, and neuronal energy metabolism, progressively reducing the ability of neural circuits to maintain homeostasis under additional metabolic or vascular stress [114,115].

Clinically, patients with CKD, particularly those receiving dialysis, often show elevated circulating IL-1β, IL-6, TNF-α, and related mediators associated with cognitive impairment and putative markers of neuroinflammation [117,118,119,120,121].

Additional molecules may also contribute to CKD-associated neuroimmune dysfunction. Elevated circulating S100B, a marker associated with glial and BBB dysfunction, has been linked to cognitive impairment in patients with ESKD; however, its causal and mechanistic role remains uncertain [122]. In contrast, fibrinogen has been more directly implicated in neuroinflammatory amplification following BBB leakage, through activation of microglia and astrocytes, oxidative stress, and subsequent synaptic and neuronal injury [123,124,125]. Clinical studies in non-CKD neurodegenerative populations have additionally demonstrated associations between circulating fibrinogen and markers of neurovascular and neurodegenerative pathology, although these findings remain predominantly observational [126]. Together with broader evidence that blood-borne systemic factors can influence brain homeostasis, aging, and neurodegeneration [127], these observations suggest that multiple systemic inflammatory pathways may contribute to chronic neuroimmune dysfunction in CKD. Nevertheless, CKD-specific clinical evidence remains limited and largely associative, while direct demonstrations of ongoing neuroinflammation in living patients remain methodologically challenging. Future studies integrating fluid biomarkers with advanced neuroimaging and functional assessment of neurovascular integrity will be required to establish the temporal relationship among systemic inflammation, neuroglial activation, and cognitive decline.

Overall, reciprocal signaling among endothelial cells, microglia, astrocytes, and neurons provides a plausible mechanism for CKD-associated neuroinflammation, but longitudinal studies are needed to determine whether these alterations predict cognitive decline or acute encephalopathic decompensation.

## 7. Redox Imbalance and Mitochondrial Dysfunction: A Convergent Pathway of Neuronal Vulnerability

Oxidative stress and mitochondrial dysfunction form closely interconnected pathways through which CKD-associated systemic disturbances may promote neuronal and synaptic injury [40,128,129]. Redox imbalance, impaired bioenergetics, organelle stress, defective proteostasis, and inflammatory signaling may reinforce one another, reducing cerebral resilience [130,131,132].

### 7.1. CKD-Associated Systemic Redox Imbalance

CKD is characterized by excess reactive oxygen and nitrogen species relative to antioxidant capacity. Uremic toxin retention, inflammation, endothelial injury, metabolic abnormalities, and depleted antioxidant defenses generate a systemic pro-oxidative environment that can extend to the brain.

In 5/6-nephrectomized rats, cerebral NF-κB activation and pro-oxidant/inflammatory signaling were accompanied by reduced nuclear NRF2 translocation and lower glutathione peroxidase, catalase, and heme oxygenase-1 expression [128]. Thus, CKD-associated cerebral oxidative stress reflects both increased oxidant production and impaired endogenous antioxidant responses.

Redox imbalance also affects the cerebral vasculature. Reduced eNOS expression, increased nitrosative stress, and lower ZO-1, occludin, and JAM-1 are consistent with impaired nitric oxide signaling and BBB injury [128]. Clinical biomarker studies support a systemic inflammatory, endothelial, and prothrombotic phenotype in stage 5 CKD, although they do not directly demonstrate cerebral barrier damage [133].

Oxidative stress can both damage mitochondria and be amplified by mitochondrial dysfunction, creating a feed-forward cycle of impaired bioenergetics and ROS production. Defective microglial clearance may further sustain this redox-inflammatory environment, although this has mainly been shown in CKD models with superimposed ischemic injury [113].

### 7.2. Mitochondrial Bioenergetic Failure and Quality-Control Dysfunction

Neurons are particularly vulnerable to mitochondrial injury because of their high energy requirements and dependence on oxidative phosphorylation to maintain membrane potential, ion gradients, neurotransmitter release, calcium buffering, and synaptic plasticity [130,132].

Direct experimental evidence supports cerebral mitochondrial dysfunction in CKD. Adenine-induced CKD reduces respiratory-chain activity in selected brain regions and is accompanied by oxidative stress, cholinergic dysfunction, behavioral abnormalities, dendritic spine loss, impaired hippocampal arborization, and astrocytic activation [40].

More generally, mitochondrial oxidative injury damages mitochondrial DNA, membrane phospholipids, and respiratory-chain proteins, further reducing oxidative phosphorylation and promoting additional ROS formation. The broader neurodegeneration literature similarly identifies mitochondrial bioenergetic failure, redox imbalance, and defective quality control as convergent drivers of cognitive and neuronal dysfunction [134,135]. Mitochondrial dynamics and quality-control pathways are also essential for neuronal viability. Disturbances in mitochondrial fission, fusion, transport, and mitophagy can permit the accumulation of dysfunctional organelles and reduce mitochondrial delivery to distal neuronal processes, with adverse consequences for local ATP supply, calcium buffering, and synaptic function [130,132,136]. However, direct evidence that CKD induces each of these specific quality-control abnormalities in the brain remains limited.

The absence of adenine or 2,8-dihydroxyadenine crystal deposition in the brains of adenine-treated CKD mice supports a systemic, rather than directly crystal-associated, origin of the neurological abnormalities [40].

### 7.3. Endoplasmic Reticulum Stress and Impaired Proteostasis

Mitochondrial dysfunction also interacts with endoplasmic reticulum (ER) stress. Mitochondria–ER contact sites regulate calcium transfer, lipid metabolism, energy production, mitochondrial dynamics, and cell-death signaling; dysfunction of either organelle may therefore amplify cellular injury [131,132].

In a 5/6 nephrectomy CKD model, increased hippocampal hydroxynonenal (HNE)-protein adducts and glucose-regulated protein 78 indicated concurrent oxidative and ER stress and correlated with serum blood urea nitrogen and creatinine [129]. C/EBP homologous protein (CHOP) expression was not increased, suggesting activation of an adaptive unfolded protein response without clear engagement of the downstream ER stress-mediated apoptotic pathway at the examined time point.

Persistent ER stress may nevertheless impair protein folding, calcium homeostasis, and proteostasis, further compromising mitochondrial function and neuronal capacity to maintain synaptic integrity.

### 7.4. Uremic Toxins and RAS Activation as Upstream Triggers and Amplifiers

Protein-bound uremic toxins represent important CKD-specific triggers of cerebral oxidative, inflammatory, and metabolic dysfunction. In primary human astrocytes, IS increases ROS production, induces mitochondrial membrane depolarization and apoptosis, and produces widespread transcriptional changes involving oxidative stress, NRF2, MAPK, and endoplasmic-reticulum protein-processing pathways [41]. Prolonged IS exposure also suppresses astrocytic glycolytic activity and ATP production by reducing the expression of glycolysis-related enzymes and pyruvate dehydrogenase, including HK1, PFKP, PKM2, LDHA, and PDH [42]. Because astrocytes provide metabolic and trophic support to neurons and contribute to glutamate recycling and extracellular ion homeostasis, combined impairment of glycolysis and mitochondrial function may substantially reduce astrocyte-mediated neuronal support [114,115].

Combined exposure to retained metabolites can produce greater neuronal toxicity than individual solutes. A mixture of IS, indole, indole-3-acetate, and methylglyoxal reduced HT-22 neuronal viability and glutathione at lower concentrations than generally required for individual compounds [36].

In unilateral-nephrectomy models with experimental IS or PCS exposure, AST-120 treatment reduced cerebral oxidative and inflammatory abnormalities and improved cognitive or behavioral outcomes [37,39]. As AST-120 reduces multiple gut-derived uremic solutes, these findings are best interpreted as supporting a broader reduction in uremic toxin burden rather than an effect attributable to a single toxin.

Renin–angiotensin system (RAS) activation may further amplify this injury. Angiotensin II type 1 receptor blockade reduces hippocampal oxidative stress and preserves synaptic plasticity in experimental kidney injury [137]. In 5XFAD mice with adenine-induced CKD, olmesartan improved cognition and attenuated hippocampal oxidative stress and BBB disruption without substantially improving renal dysfunction [138], supporting RAS as an amplifier rather than a primary independent mechanism.

### 7.5. Loss of Neuronal Reserve

Experimental findings suggest that cellular injury can precede overt behavioral deficits. Adenine-induced CKD rats showed hippocampal neuronal pyknosis and oxidative stress despite no significant Morris water maze impairment [36], while CKD accelerated cognitive impairment in 5XFAD but not wild-type mice, indicating greater sensitivity in a vulnerable brain [138].

**Figure 2:**
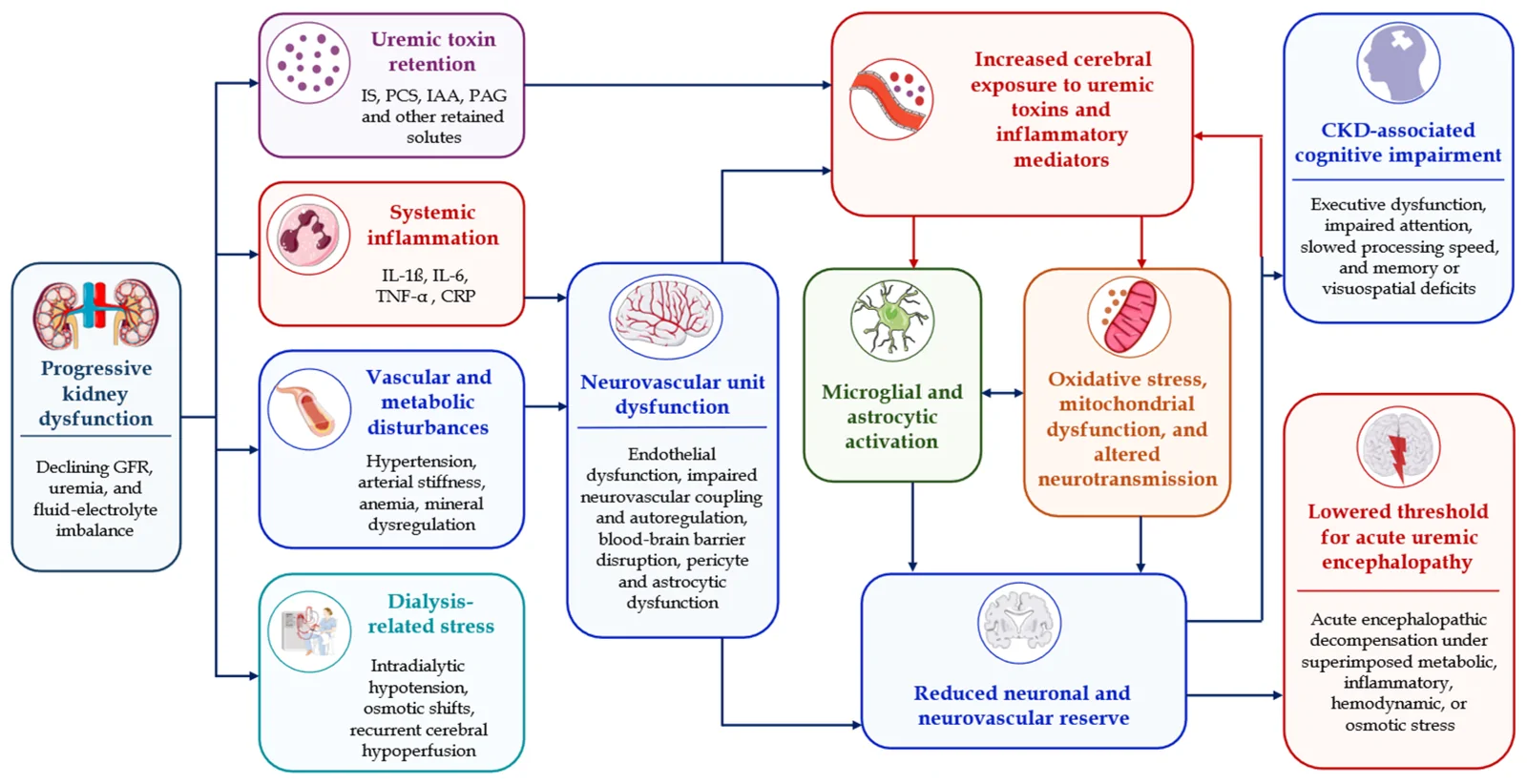
Interconnected pathways linking progressive kidney dysfunction to brain injury. Declining kidney function promotes uremic toxin retention, systemic inflammation, vascular and metabolic disturbances, and dialysis-related physiological stress. These factors disrupt the neurovascular unit, increase cerebral exposure to uremic toxins and inflammatory mediators, and promote reciprocal glial activation, oxidative stress, mitochondrial dysfunction, and altered neurotransmission. Their interaction progressively impairs cerebral homeostasis and reduces neuronal and neurovascular reserve, contributing to chronic cognitive impairment and lowering the threshold for acute uremic encephalopathy. CKD, chronic kidney disease; CRP, C-reactive protein; GFR, glomerular filtration rate; IAA, indole-3-acetic acid; IL, interleukin; IS, indoxyl sulfate; PAG, phenylacetylglutamine; PCS, p-cresyl sulfate; TNF-α, tumor necrosis factor-α;. Created using Microsoft PowerPoint and elements from Servier Medical Art (https://smart.servier.com), adapted under the Creative Commons Attribution 4.0 International License (CC BY 4.0; https://creativecommons.org/licenses/by/4.0/ (accessed on 21 July 2026)).

**Figure 3:**
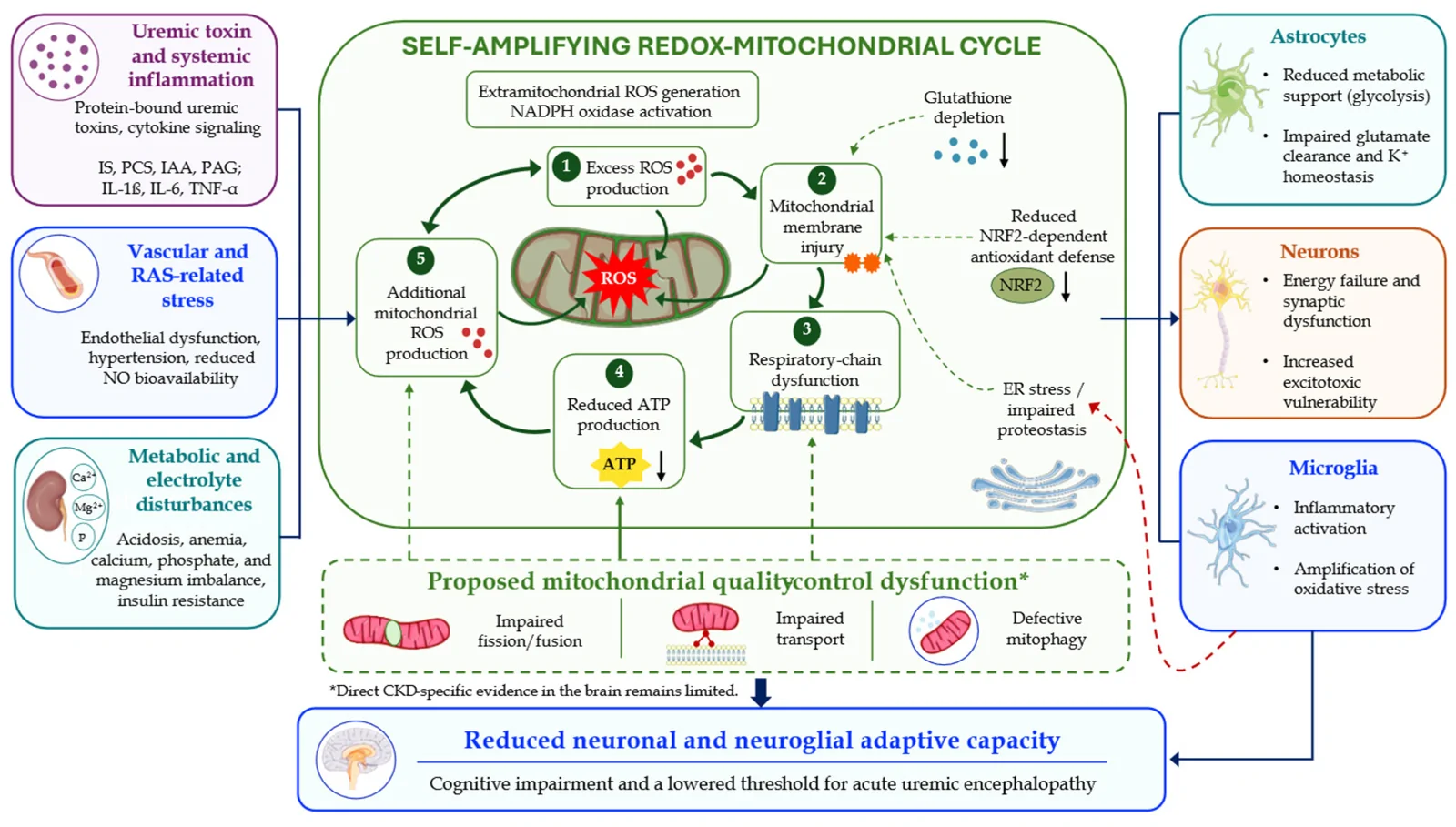
Redox imbalance and mitochondrial dysfunction as convergent mechanisms of neuronal vulnerability in CKD. Uremic toxins, systemic inflammation, vascular and renin–angiotensin system-related stress, and metabolic disturbances promote excess oxidant production, impaired antioxidant defense, mitochondrial injury, respiratory-chain dysfunction, and reduced ATP generation. These mutually reinforcing processes impair astrocytic metabolic and homeostatic support, neuronal energy and synaptic function, and microglial inflammatory regulation. Disturbances in mitochondrial fission, fusion, transport, and mitophagy are presented as proposed contributing mechanisms because direct CKD-specific evidence in the brain remains limited. Taken together, redox, mitochondrial, and cellular dysfunction may reduce neuronal and neuroglial adaptive capacity, contributing to cognitive impairment and lowering the threshold for acute uremic encephalopathy. ATP, adenosine triphosphate; CKD, chronic kidney disease; ER, endoplasmic reticulum; IL, interleukin; NADPH, nicotinamide adenine dinucleotide phosphate; NRF2, nuclear factor erythroid 2-related factor 2; RAS, renin–angiotensin system; ROS, reactive oxygen species; TNF-α, tumor necrosis factor-α. Created using Microsoft PowerPoint and elements from Servier Medical Art (https://smart.servier.com), adapted under the Creative Commons Attribution 4.0 International License (CC BY 4.0; https://creativecommons.org/licenses/by/4.0/ (accessed on 21 July 2026)).

With sustained kidney dysfunction, mitochondrial impairment, weakened antioxidant defenses, disturbed proteostasis, and reduced astrocytic metabolic support may progressively diminish neuronal and neurovascular reserve, increasing susceptibility to superimposed uremic, metabolic, inflammatory, or hemodynamic stress (Figure 2 and Figure 3).

## 8. Uremic Encephalopathy: Clinical Expression and Mechanisms

### 8.1. Clinical Features and Diagnostic Considerations

UE is an acute or subacute syndrome of diffuse cerebral dysfunction occurring in the setting of severe kidney failure and substantial accumulation of retained uremic solutes. Its clinical expression is heterogeneous and may range from impaired attention, apathy, irritability, and fluctuating cognition to disorientation, delirium, somnolence, seizures, and coma. Motor manifestations may include asterixis, tremor, myoclonus, and other abnormalities of motor control [12,139,140].

No single neurological sign, biochemical threshold, electroencephalographic pattern, or neuroimaging abnormality is diagnostic of UE. The diagnosis therefore remains primarily clinical and is established in the appropriate context of advanced kidney dysfunction after exclusion of alternative metabolic, toxic, vascular, infectious, and structural causes of encephalopathy [12]. Improvement in neurological status after effective kidney replacement therapy provides important supportive evidence and may contribute retrospectively to the diagnosis, although the degree and rate of recovery vary among patients [12,141]. Persistent or disproportionate impairment of consciousness, particularly in the presence of myoclonus or subtle motor phenomena, should also prompt consideration of electrographic seizures or non-convulsive status epilepticus [140].

### 8.2. Electroencephalographic Features

Electroencephalographic abnormalities are well recognized in UE, although no EEG pattern is specific for uremia. The most consistently reported findings include diffuse slowing of background activity, attenuation or slowing of the normal alpha rhythm, and increased theta and delta activity, reflecting generalized cerebral dysfunction rather than a disease-specific electrophysiological signature [142,143,144,145]. Quantitative EEG analyses have similarly demonstrated increased low-frequency activity in uremic states, supporting the potential role of EEG in the objective assessment of cerebral dysfunction.

Early clinical studies suggested that EEG abnormalities may evolve in parallel with the severity of UE. Noriega-Sanchez et al. described sequential clinical and electroencephalographic changes in patients with progressive uremic encephalopathy, whereas Kiley et al. and Bourne and Teschan demonstrated that quantitative EEG analysis can characterize cerebral dysfunction associated with increasing uremic burden [142,144,145]. However, much of this direct evidence derives from relatively small and older clinical studies and should therefore be interpreted cautiously.

Triphasic waves represent another recognized EEG pattern in toxic-metabolic encephalopathies. Typical triphasic waves consist of bilateral, synchronous, predominantly fronto-central complexes, often occurring at approximately 1.5–2 Hz and displaying a fronto-occipital lag [146]. They may occur in hepatic, uremic, and sepsis-associated encephalopathies but are not specific for uremia. Severe uremia may also be associated with myoclonic, focal, or generalized seizures and with convulsive or non-convulsive status epilepticus [140]. Accordingly, persistent or disproportionate impairment of consciousness in suspected UE should prompt consideration of continuous EEG monitoring when electrographic seizure activity is a concern.

Electrophysiological abnormalities may improve following correction of the uremic state. In the case series reported by Yanai et al., neurological recovery following hemodialysis was accompanied by reduction in diffuse slow-wave activity and re-emergence of a predominantly alpha background [141]. EEG therefore provides supportive rather than diagnostic evidence of UE, with particular value in documenting diffuse cerebral dysfunction, identifying concomitant seizures or non-convulsive status epilepticus, and monitoring neurophysiological improvement after correction of severe uremia. Related but less severe EEG abnormalities have also been reported in CKD-associated mild cognitive impairment, although these findings should not be interpreted as UE-specific markers [147].

### 8.3. Magnetic Resonance Imaging Features

Magnetic resonance imaging (MRI) provides supportive information in patients with suspected UE, particularly when alternative structural, vascular, or metabolic causes of encephalopathy must be excluded. However, UE has no single diagnostic neuroimaging signature. Reported abnormalities are heterogeneous and can be broadly grouped into basal ganglia, cortical or subcortical, and white-matter patterns [148,149,150]. Diffusion-weighted imaging (DWI) and apparent diffusion coefficient (ADC) mapping may help characterize predominantly vasogenic, cytotoxic, or mixed components of cerebral edema. The heterogeneity of these findings is illustrated by Sina et al., in whose prospective cohort none of 20 patients with clinically diagnosed UE showed basal ganglia involvement or the lentiform fork sign, whereas white-matter abnormalities were common [149].

Basal ganglia involvement is one of the best-described MRI phenotypes of UE and is typically bilateral and relatively symmetric, with T2-weighted/fluid-attenuated inversion recovery (FLAIR) hyperintensity involving the lentiform nuclei and adjacent structures [148,151,152]. This pattern has been described particularly in diabetic uremia and may accompany extrapyramidal manifestations such as parkinsonism, dyskinesia, or chorea. Diffusion-weighted imaging (DWI) findings are variable, and both vasogenic and cytotoxic components have been reported, suggesting heterogeneity in the severity and mechanisms of tissue injury [152]. Similar bilateral basal ganglia abnormalities have also been reported in non-diabetic patients receiving hemodialysis [153].

A particularly recognizable pattern is the lentiform fork sign (LFS), consisting of a T2/FLAIR-hyperintense rim outlining the lentiform nucleus and adjacent capsular structures. Although repeatedly described in UE, particularly in association with severe metabolic acidosis, the LFS is not pathognomonic for uremia. It has been reported in other metabolic and toxic conditions and in posterior reversible encephalopathy syndrome (PRES) without renal failure [154,155]. Conversely, its absence does not exclude UE [149]. The LFS should therefore be regarded as a characteristic but nonspecific imaging pattern that requires interpretation within the clinical and metabolic context.

Cortical and subcortical abnormalities may produce a posterior-predominant appearance resembling posterior reversible encephalopathy syndrome (PRES) [149,156]. PRES should not, however, be regarded as synonymous with UE. Severe or fluctuating hypertension, volume disturbances, endothelial dysfunction, and kidney failure may predispose patients to PRES independently of uremic neurotoxicity, making it an important overlapping or alternative cause of acute encephalopathy [157,158]. White-matter, internal-capsular, and, more rarely, brainstem abnormalities have also been reported, further illustrating the radiological heterogeneity of UE.

Partial or complete radiological regression after dialysis or correction of renal and metabolic derangements has been documented in several reports and small series [148,152,153]. Nevertheless, radiological reversibility is not universal. MRI should therefore be regarded as an adjunct to clinical and biochemical assessment rather than a diagnostic test for UE. Its principal value lies in characterizing cerebral injury, excluding competing neurological disorders, and, when follow-up imaging is available, documenting potential reversibility.

### 8.4. Differential Diagnosis and Dialysis-Related Encephalopathies

UE remains primarily a clinical diagnosis of exclusion because its neurological manifestations, EEG abnormalities, and neuroimaging findings are not individually specific. In patients with advanced kidney failure who develop acute or subacute encephalopathy, potentially reversible metabolic, toxic, vascular, infectious, and nutritional causes should therefore be actively considered. These include severe electrolyte and acid–base disturbances, hypo- or hyperglycemia, hypertensive encephalopathy and PRES, cerebrovascular events, sepsis-associated encephalopathy, central nervous system infection, and neurotoxicity related to medications whose clearance is impaired in kidney failure [12,140].

Thiamine deficiency and Wernicke encephalopathy should also be considered, particularly in malnourished, critically ill, or dialysis-treated patients with kidney disease, in whom reduced nutritional intake, gastrointestinal symptoms, and losses associated with kidney replacement therapy may increase the risk of deficiency [159]. Because Wernicke encephalopathy may present predominantly with altered mental status and does not necessarily manifest with the complete classical triad, it may be mistaken for other metabolic encephalopathies, including UE [159]. Historically, aluminum-associated encephalopathy represented an important neurological complication in dialysis populations, although its relevance has markedly declined with contemporary dialysis practice [12]. Multiple mechanisms may also coexist in patients with advanced kidney failure, complicating attribution of neurological dysfunction to a single cause.

Dialysis disequilibrium syndrome (DDS) should be distinguished from UE despite partially overlapping manifestations. Whereas UE reflects cerebral dysfunction associated with severe uremia and generally improves after effective kidney replacement therapy, DDS develops during or shortly after dialysis, particularly when rapid solute removal occurs in markedly uremic patients. Rapid reduction in plasma urea and osmolality may generate an osmotic gradient favoring cerebral water influx and cerebral edema, resulting in headache, nausea, confusion, seizures, and, in severe cases, coma [12,140]. Quantitative MRI studies have also demonstrated transient increases in total and gray-matter brain volume during hemodialysis, supporting acute cerebral water shifts during rapid solute removal [160]. Thus, dialysis may reverse UE while, under specific conditions, rapid dialysis itself may precipitate a distinct osmotic encephalopathy.

### 8.5. Acute Neurotoxic and Metabolic Burden

Direct human mechanistic evidence specific to uremic encephalopathy remains limited. Consequently, current concepts of its pathophysiology are derived from a combination of clinical observations in severe uremia, biochemical studies, and experimental CKD or toxin-exposure models whose relevance to overt human UE varies [12]. In advanced kidney failure, a combined neurotoxic, metabolic, inflammatory, and hemodynamic burden may converge on disturbances in neurotransmission, cellular metabolism, neuronal excitability, glial support, and BBB function, potentially overwhelming cerebral compensatory mechanisms [12].

Advanced uremia is characterized by systemic retention of multiple solutes whose cerebral exposure depends on physicochemical properties, protein binding, BBB integrity, and active transport mechanisms. These compounds include small water-soluble solutes, protein-bound toxins, and middle molecules and may interact through pathways involving oxidative stress, mitochondrial dysfunction, endothelial injury, neuroinflammation, and altered neuronal signaling [12,21].

Current evidence does not identify a single uremic neurotoxin sufficient to explain UE. Encephalopathic manifestations are more plausibly related to the combined effects of several retained compounds, including guanidino compounds, IS, PCS, homocysteine, β_2_-microglobulin, and other middle molecules [12].

Experimental studies demonstrate that individual retained solutes can exert direct neurobiological effects. Chronic IS exposure in rats resulted in cerebral toxin accumulation accompanied by region-specific neurochemical changes, increased lipid peroxidation, and behavioral and cognitive abnormalities [38]. However, such toxin-exposure models do not reproduce the complex metabolic milieu of advanced human uremia and should therefore be interpreted as mechanistic support rather than direct models of UE. In vitro studies similarly suggest that combinations of uremic toxins may exert greater neuronal toxicity than individual solutes, supporting the concept that neurological dysfunction may reflect cumulative or interacting toxin effects rather than a single dominant compound [36].

Cerebral exposure to retained solutes is further influenced by BBB transport and barrier integrity. Organic anion transporters mediate the brain-to-blood efflux of several uremic solutes, including 3-carboxy-4-methyl-5-propyl-2-furanpropionic acid (CMPF), hippurate, and indoleacetate. Experimental inhibition of these transport pathways reduces toxin efflux [161]. In parallel, BBB disruption demonstrated in experimental CKD may facilitate neural exposure to circulating toxins and inflammatory mediators [12,128]. Although these mechanisms have not been directly established as causal pathways in human UE, they provide a plausible biological basis by which severe uremia could increase cerebral neurotoxic burden and contribute to acute encephalopathic decompensation.

### 8.6. Neurotransmitter Dysregulation, Network Dysfunction, and Potential Reversibility

Altered neuronal excitability represents a plausible downstream consequence of the neurotoxic and metabolic environment of advanced uremia. Among the best-characterized mechanisms is disruption of the balance between excitatory and inhibitory neurotransmission. Guanidinosuccinic acid can enhance N-methyl-D-aspartate (NMDA) receptor-mediated excitation, whereas several other uremic guanidino compounds inhibit γ-aminobutyric acid type A (GABA_A) receptor-mediated transmission. Experimental exposure of hippocampal preparations to guanidine compounds or serum from patients undergoing dialysis has also been associated with enhanced glutamatergic transmission involving presynaptic glutamate release and α-amino-3-hydroxy-5-methyl-4-isoxazolepropionic acid/N-methyl-D-aspartate (AMPA/NMDA) receptor-mediated responses [44,162]. The resulting shift toward excitatory dominance may promote neuronal hyperexcitability and excitotoxic vulnerability, contributing particularly to myoclonus and seizures. A broader contribution to delirium and altered consciousness is possible but remains speculative and requires confirmation in in vivo and clinical studies.

Additional experimental evidence suggests that uric acid, IS, and methylguanidine can activate bulbospinal neurons within the rostral ventrolateral medulla through toxin-specific transport mechanisms and NADPH oxidase-dependent oxidative stress [163]. Although this experimental system does not reproduce UE, it does provide evidence that retained solutes are capable of altering central neuronal activity, but should not be interpreted as proof of a specific mechanism of human UE.

Monoaminergic signaling may also be affected by CKD and selected retained solutes, particularly IS, but available findings are heterogeneous, and the contribution of dopaminergic or other monoaminergic abnormalities to overt UE remains uncertain [38,164].

At the systems level, the combined effects of altered neurotransmission, metabolic disturbance, oxidative stress, and neurotoxic exposure may produce diffuse cortical and subcortical network dysfunction rather than selective impairment of a single neuronal population. This interpretation is consistent with the global neurological phenotype of UE and with the diffuse EEG abnormalities and heterogeneous, often bilateral MRI patterns described above.

Taken together, these mechanisms may produce diffuse cortical and subcortical network dysfunction rather than selective impairment of a single neuronal population, consistent with the widespread EEG abnormalities and heterogeneous bilateral MRI patterns described above. Improvement in consciousness and neurological manifestations following effective kidney replacement therapy supports a substantial functional and potentially reversible component to cerebral dysfunction [12,141]. Nevertheless, recovery may be incomplete, particularly when UE occurs on a background of pre-existing cerebrovascular, neurodegenerative, or other structural cerebral injury [12]. UE may therefore be conceptualized as an acute or subacute state of diffuse, potentially reversible cerebral dysfunction superimposed on a variable background of chronic neuronal and neurovascular vulnerability. This interaction between acute physiological disturbance and pre-existing cerebral susceptibility provides the rationale for the reserve–threshold model discussed in the following section.

## 9. From Cognitive Impairment to Uremic Encephalopathy: A Proposed Pathophysiological Continuum

CKD-associated cognitive impairment and UE are clinically distinct but biologically interconnected manifestations of CKD-associated brain dysfunction. We propose a non-linear reserve–threshold model in which cumulative cerebral injury progressively reduces adaptive capacity, while UE may emerge when acute uremic or systemic stress exceeds the remaining neuronal and neurovascular reserve. The general concept that acute cerebral dysfunction may result from the interaction between baseline vulnerability and superimposed precipitating factors has an important precedent in the vulnerability-precipitant model of delirium proposed by Inouye and Charpentier [165]. The present reserve–threshold model builds on this principle but applies it specifically to CKD-associated brain dysfunction. In this context, “reserve” refers to neuronal and neurovascular adaptive capacity that may be progressively reduced by CKD-associated toxic, vascular, inflammatory, metabolic, and synaptic injury. Acute increases in uremic burden and superimposed metabolic, inflammatory, hemodynamic, or osmotic disturbances are conceptualized as threshold-crossing stressors that may precipitate overt UE. Thus, the proposed model is not intended as a new general model of delirium, but as a CKD-specific mechanistic extension linking chronic cerebral vulnerability with acute uremic decompensation.

### 9.1. Accumulation of Chronic Cerebral Vulnerability

In earlier stages of CKD, CKD-associated cerebral abnormalities may be present without overt global cerebral dysfunction. Compensatory mechanisms may initially preserve cerebral homeostasis. With progression, structural lesions such as white matter injury and microbleeds may reduce anatomical reserve, while impaired neurovascular, glial, and metabolic function may progressively reduce functional reserves [14,17,18,19,20,32,33,40,45,52,58,59,60,61,62,63,64,76,77,128,138].

This cumulative burden may contribute to executive, attentional, and processing-speed deficits. Cognitive dysfunction may contain both relatively fixed structural components, such as CSVD and neuronal loss, and dynamic components, including toxin-related signaling, metabolic inhibition, altered perfusion, and glial activation, which may vary with kidney function, dialysis, inflammation, and hemodynamics [18,27,64,90].

### 9.2. A Threshold Model of Acute Encephalopathic Decompensation

As cerebral reserves decline, severe uremic and systemic stress may disrupt neuronal, metabolic, and neurovascular homeostasis beyond the remaining compensatory capacity [12,38,44,58,59,60,61,62,63,64,70,71,72,73,74,161,162].

Within this model, UE is proposed to emerge when combined uremic neurotoxic and metabolic stress exceeds the remaining capacity for cerebral adaptation. The transition is better viewed as a state change in network function than as the final step of a uniform neurodegenerative sequence. Improvement after correction of uremia supports a substantial functional component, although recovery may be incomplete when chronic cerebrovascular or neurodegenerative pathology has already reduced reserves [12,13,141].

Electrolyte and acid–base disturbances, systemic inflammation, hemodynamic instability, and dialysis-related osmotic stress may further lower the threshold for acute deterioration. Thus, the proposed continuum reflects the interaction between chronic loss of cerebral reserves and the intensity of superimposed acute stress.

### 9.3. Non-Linearity and Interindividual Heterogeneity

The continuum is pathophysiological rather than obligatorily chronological. Some patients may develop chronic cognitive impairment through predominantly vascular and structural pathways without UE, whereas others may develop UE abruptly during advanced kidney failure with little recognized prior cognitive decline. Pre-existing impairment may also remain clinically unrecognized [7,19,20,46,51].

Susceptibility likely varies with age, underlying cerebral disease, comorbidity, residual kidney function, toxin burden, dialysis-related factors, and hemodynamic stability. Consequently, similar biochemical degrees of kidney failure may produce different neurological phenotypes, and no single urea, creatinine, or toxin concentration currently defines the transition to UE [12,13].

### 9.4. Testable Predictions of the Proposed Model

The model generates several testable predictions. Patients with lower baseline cognition, greater CSVD, impaired BBB integrity, or reduced neurovascular responsiveness may be more vulnerable to neurological deterioration during advanced uremia. Serial changes in protein-bound toxins, inflammatory markers, cerebral perfusion, BBB permeability, and glial or neuronal injury markers may track neurological trajectories better than isolated urea or creatinine measurements [18,25,26,27,58,59,60,61,62,63,64,89,90]. Interventions that reduce uremic burden or stabilize neurovascular and metabolic function may be most effective before extensive structural injury, while patients recovering from UE may retain cognitive, vascular, or imaging abnormalities reflecting pre-existing vulnerability.

Prospective studies combining standardized neurological and cognitive assessment with neurovascular imaging, uremic toxin measurements, and relevant kidney- and dialysis-related variables will be required to determine whether reduced cerebral reserves predict encephalopathic decompensation and whether preservation of neurovascular and metabolic function modifies the neurological course of CKD.

### 9.5. Limitations

Several limitations of both the available evidence and the present review should be acknowledged. Much of the available human evidence is observational and frequently cross-sectional, so reverse causation and residual confounding cannot be fully excluded. Human mechanistic data specific to uremic encephalopathy remain scarce, and several proposed pathophysiological pathways are supported primarily by animal or cellular studies and have not yet been confirmed in humans. Although these experimental models provide important mechanistic insights, rodent models do not fully reproduce the complexity of advanced human uremia and therefore have limited construct validity for some aspects of UE. Direct longitudinal evidence validating the proposed reserve–threshold model, including the proposed transition from chronic cerebral vulnerability to overt uremic encephalopathy, is also lacking.

In addition, cognitive changes after kidney transplantation were not examined in detail, although transplantation and restoration of kidney function may provide important information regarding the reversibility of CKD-associated cognitive dysfunction. Similarly, the potential influence of age at CKD onset was beyond the scope of this review. Pediatric-onset CKD may involve distinct neurodevelopmental consequences and mechanisms and should be specifically addressed in future studies.

Finally, this narrative review used an iterative rather than systematic search strategy, and relevant studies may consequently have been missed. These limitations should be considered when interpreting the proposed model, which is intended as a hypothesis-generating conceptual framework rather than as a clinically validated staging system.

## 10. Clinical Implications and Future Directions

This model has several potential clinical implications, although its predictive value has not yet been established. Cognitive screening may help identify pre-existing vulnerability or reduced cerebral reserve, particularly in older adults, patients with advanced CKD, and those with substantial cerebrovascular disease or recurrent intradialytic hypotension. Preventive strategies should address modifiable vascular and metabolic risk factors, preserve residual kidney function where possible, minimize avoidable dialysis-related hemodynamic instability, and improve early recognition of acute neurological deterioration in advanced kidney failure.

Mechanism-based interventions remain largely preclinical. Experimental reductions in the effects of protein-bound uremic toxins have attenuated oxidative stress, neuroinflammatory activation, neuronal injury, and cognitive or behavioral abnormalities in toxin-exposure models involving IS and PCS [37,39]. Other experimental approaches targeting redox and gasotransmitter signaling have improved cognitive performance and reduced neuronal injury through pathways involving nitric oxide, hydrogen sulfide, cystathionine β-synthase activity, and NRF2/HO-1 signaling [166,167]. These findings highlight the need to further explore strategies that reduce the burden of poorly cleared solutes, restore antioxidant and mitochondrial capacity, preserve BBB and neurovascular function, correct metabolic disturbances, and limit neuroinflammatory signaling. Such interventions may be most effective before extensive and potentially irreversible structural injury develops, although the optimal therapeutic window remains unknown. Clinical translation will require approaches that target upstream CKD-specific drivers while accounting for the parallel contributions of vascular risk factors, aging, stroke, and neurodegenerative disease.

Several major knowledge gaps remain. Longitudinal studies are needed to determine whether specific patterns or trajectories of cognitive impairment predict subsequent UE and to establish the temporal relationships among toxin accumulation, BBB permeability, neuroinflammation, mitochondrial injury, and cognitive decline. Standardized cognitive phenotyping, quantitative BBB-permeability and cerebral-perfusion imaging, repeated measurement of free and total concentrations of protein-bound uremic toxins, serial assessment of residual kidney function, and integration of fluid biomarkers with dialysis and hemodynamic parameters could help clarify causal pathways. Future studies should also identify which mechanisms contribute to chronic cognitive impairment, which specifically precipitate altered consciousness, myoclonus, seizures, and other defining manifestations of UE, and whether preservation of cerebral reserves modifies the risk or severity of acute decompensation.

## 11. Conclusions

CKD-associated cognitive impairment and UE are clinically distinct syndromes that may share a common background of reduced cerebral resilience.

The CKD-specific reserve–threshold model integrates chronic cerebral vulnerability with superimposed acute uremic and systemic stress without implying an obligatory progression from cognitive impairment to UE.

This hypothesis-generating model requires prospective validation. Distinguishing mechanisms that progressively reduce cerebral reserves from those that precipitate overt UE may ultimately improve identification of vulnerable patients and inform preventive interventions before acute encephalopathic decompensation occurs.

## Data Availability

No new data were created or analyzed in this study. Data sharing is not applicable to this article.
